# Full-Length Human Placental sFlt-1-e15a Isoform Induces Distinct Maternal Phenotypes of Preeclampsia in Mice

**DOI:** 10.1371/journal.pone.0119547

**Published:** 2015-04-10

**Authors:** Gabor Szalai, Roberto Romero, Tinnakorn Chaiworapongsa, Yi Xu, Bing Wang, Hyunyoung Ahn, Zhonghui Xu, Po Jen Chiang, Birgitta Sundell, Rona Wang, Yang Jiang, Olesya Plazyo, Mary Olive, Adi L. Tarca, Zhong Dong, Faisal Qureshi, Zoltan Papp, Sonia S. Hassan, Edgar Hernandez-Andrade, Nandor Gabor Than

**Affiliations:** 1 Perinatology Research Branch, *Eunice Kennedy Shriver* National Institute of Child Health and Human Development, National Institutes of Health, Department of Health and Human Services, Bethesda, Maryland, and Detroit, Michigan, United States of America; 2 Department of Obstetrics and Gynecology, Wayne State University School of Medicine, Detroit, Michigan, United States of America; 3 Department of Pharmacology, Wayne State University School of Medicine, Detroit, Michigan, United States of America; 4 Department of Computer Science, Wayne State University, Detroit, Michigan, United States of America; 5 Center for Molecular Medicine and Genetics, Wayne State University School of Medicine, Detroit, Michigan, United States of America; 6 Department of Pathology, Wayne State University School of Medicine, Detroit, Michigan, United States of America; 7 Maternity Private Department, Kutvolgyi Clinical Block, Semmelweis University, Budapest, Hungary; 8 Institute of Enzymology, Research Centre for Natural Sciences, Hungarian Academy of Sciences, Budapest, Hungary; 9 First Department of Pathology and Experimental Cancer Research, Semmelweis University, Budapest, Hungary; Xavier Bichat Medical School, INSERM-CNRS—Université Paris Diderot, FRANCE

## Abstract

**Objective:**

Most anti-angiogenic preeclampsia models in rodents utilized the overexpression of a truncated soluble fms-like tyrosine kinase-1 (sFlt-1) not expressed in any species. Other limitations of mouse preeclampsia models included stressful blood pressure measurements and the lack of postpartum monitoring. We aimed to 1) develop a mouse model of preeclampsia by administering the most abundant human placental sFlt-1 isoform (hsFlt-1-e15a) in preeclampsia; 2) determine blood pressures in non-stressed conditions; and 3) develop a survival surgery that enables the collection of fetuses and placentas and postpartum (PP) monitoring.

**Methods:**

Pregnancy status of CD-1 mice was evaluated with high-frequency ultrasound on gestational days (GD) 6 and 7. Telemetry catheters were implanted in the carotid artery on GD7, and their positions were verified by ultrasound on GD13. Mice were injected through tail-vein with adenoviruses expressing hsFlt-1-e15a (n = 11) or green fluorescent protein (GFP; n = 9) on GD8/GD11. Placentas and pups were delivered by cesarean section on GD18 allowing PP monitoring. Urine samples were collected with cystocentesis on GD6/GD7, GD13, GD18, and PPD8, and albumin/creatinine ratios were determined. GFP and hsFlt-1-e15a expression profiles were determined by qRT-PCR. Aortic ring assays were performed to assess the effect of hsFlt-1-e15a on endothelia.

**Results:**

Ultrasound predicted pregnancy on GD7 in 97% of cases. Cesarean section survival rate was 100%. Mean arterial blood pressure was higher in hsFlt-1-e15a-treated than in GFP-treated mice (∆MAP = 13.2 mmHg, p = 0.00107; GD18). Focal glomerular changes were found in hsFlt-1-e15a -treated mice, which had higher urine albumin/creatinine ratios than controls (109.3±51.7μg/mg vs. 19.3±5.6μg/mg, p = 4.4x10^-2^; GD18). Aortic ring assays showed a 46% lesser microvessel outgrowth in hsFlt-1-e15a-treated than in GFP-treated mice (p = 1.2x10^-2^). Placental and fetal weights did not differ between the groups. One mouse with liver disease developed early-onset preeclampsia-like symptoms with intrauterine growth restriction (IUGR).

**Conclusions:**

A mouse model of late-onset preeclampsia was developed with the overexpression of hsFlt-1-e15a, verifying the *in vivo* pathologic effects of this primate-specific, predominant placental sFlt-1 isoform. HsFlt-1-e15a induced early-onset preeclampsia-like symptoms associated with IUGR in a mouse with a liver disease. Our findings support that hsFlt-1-e15a is central to the terminal pathway of preeclampsia, and it can induce the full spectrum of symptoms in this obstetrical syndrome.

## Introduction

Preeclampsia is one of the “great obstetrical syndromes” [1,2] that complicates 3–5% of pregnancies, and has a high incidence of maternal and fetal morbidity and mortality [3–10]. The clinical definition of preeclampsia includes new-onset hypertension and proteinuria after 20 weeks of gestation [4,8,11]. Based on the time of the onset of clinical symptoms, preeclampsia can be divided into early-onset (<34 weeks of gestation) and late-onset (>34 weeks of gestation) disease [8,10,12–14]. Most cases of preeclampsia are late-onset, with 75% developing after 37 weeks of gestation [8,15,16], and ~6% of cases occurring postpartum [10,17]. Late-onset preeclampsia is typically mild and occurs with less severe maternal and fetal complications than early-onset preeclampsia [12,18,19]. Early-onset preeclampsia has a higher incidence of perinatal and maternal morbidity and mortality, and a more frequent association with HELLP syndrome, intrauterine growth restriction, preterm birth, and stillbirth [5,8,10,12,18,20–32].

The origins of preeclampsia lie in the placenta, as the clinical symptoms of this syndrome usually diminish within 24–48 hours after the delivery of the placenta, and the presence of the fetus is not required for this obstetrical syndrome [11,33–38]. Preeclampsia is associated with placental histopathological lesions consistent with defective invasion of trophoblasts into the decidua and myometrium [39–47], a phenomenon that may be linked to changes in placental gene expression [48–56] and immune maladaptation [54,57–64]. As a consequence, the failure of physiologic transformation of the spiral arteries by these trophoblasts into low resistance vessels impairs the continuous blood supply of the placenta, leading to hemorheological changes, as well as endoplasmic, nitrosative, and oxidative stress of the placenta [11,37,65–69]. Early-onset preeclampsia is more frequently associated with a higher prevalence of severe placental lesions, while these lesions in late-onset preeclampsia are often mild and less frequent [10,22,49,70–74].

The current hypothesis about the development of preeclampsia implies that the placental stage is followed by a maternal stage, which includes the activation of a “terminal pathway” that will lead to the elevation of blood pressure and proteinuria [8,11,22,25,36,37,75–86]. It has been shown that the injured placenta can excessively release various factors, including syncytiotrophoblast debris, soluble anti-angiogenic molecules, cytokines, and yet unknown factors [36,49,76,77,80,81,86–95], which, in turn, lead to exaggerated maternal immune activation [36,77,96–100] and generalized endothelial cell dysfunction, including the damage of glomeruli [36,77,83,85,101–110]. As a central pathologic mechanism, an anti-angiogenic state develops in the second half of pregnancy in preeclampsia, preceding the onset of clinical symptoms [75,76,86–91,111–138]. This imbalance of angiogenic [placental growth factor (PlGF), vascular endothelial growth factor (VEGF)] and anti-angiogenic [soluble fms-like tyrosine kinase-1 (sFlt-1) and soluble endoglin (sEng)] factors has been extensively investigated [75,76,86–91,94,106,107,111–116,118–129,131–152].

**Fig 1:**
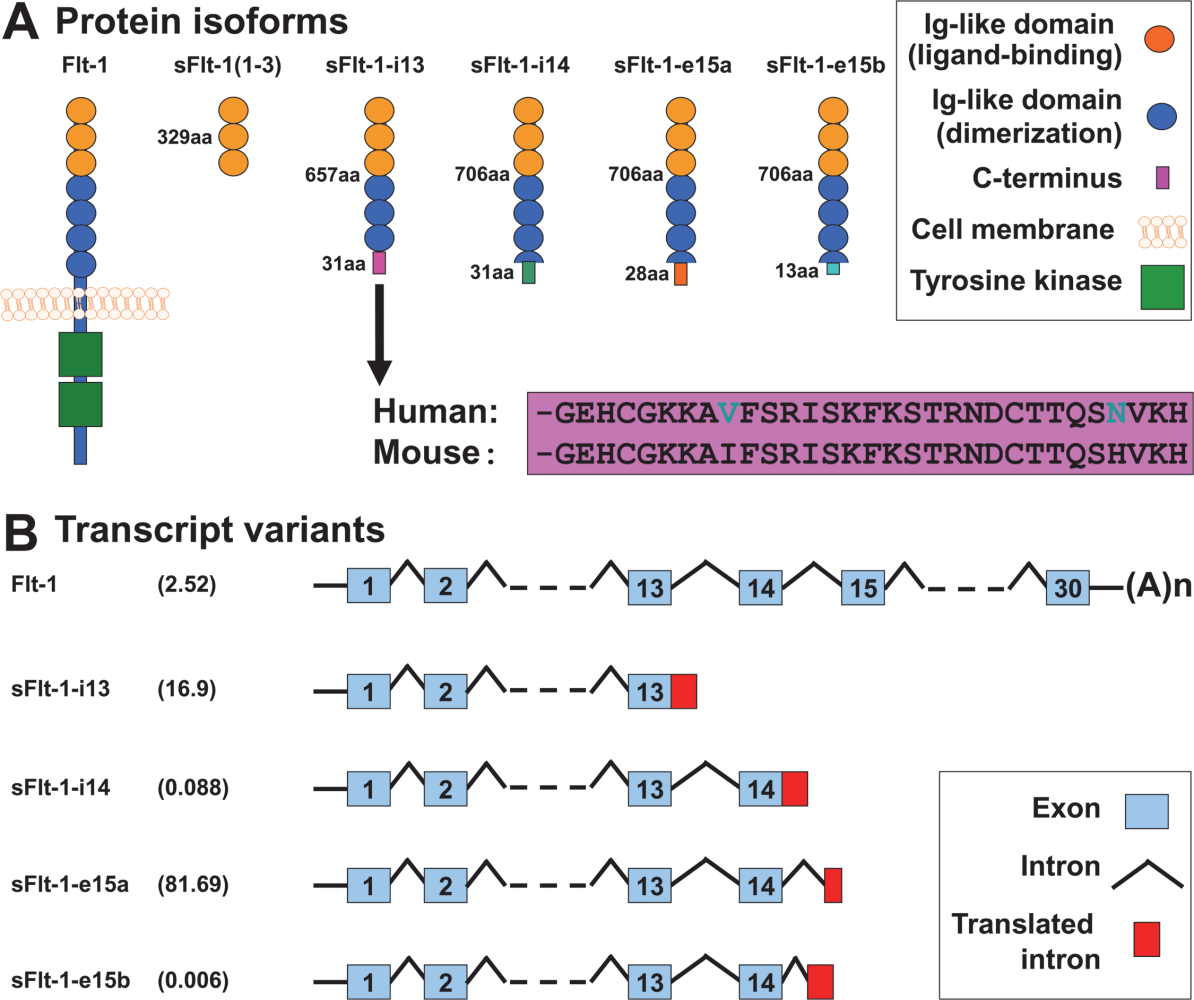
*FLT1* protein isoforms and mRNA transcript variants. (A) Flt-1 contains seven extracellular Ig-like domains and an intracellular tyrosine kinase. The first three extracellular Ig-like domains are essential for ligand-binding, while the 4–7th extracellular Ig-like domains for receptor dimerization. The truncated mouse sFlt-1 mutant [msFlt-1(1–3)] contains only 1–329 amino acids of Flt-1, corresponding to the first three Ig-like domains. Mouse and human sFlt-1-i13 contains the first six Ig-like domains corresponding to 1–657 amino acids of Flt-1, as well as a unique 31-amino-acid tail. This unique C-terminus is evolutionarily highly conserved among mammals; the mouse and human amino acid sequences of this tail are only different in two positions (shown with blue letters). Among the human placental expressed sFlt-1 isoforms, hsFlt-1-i14, hsFlt-1-e15a and hsFlt-1-e15b diverge from Flt-1 after amino acid 706, and contain a 31-, 28- and 13-amino-acid unique tails, respectively. (B) Among the placental expressed *FLT1* transcripts, the abundance of the mRNA encoding for the transmembrane receptor is about 2.5% in preeclampsia. *FLT1* transcript expression data was retrieved from Jebbink et al. and is shown as transcript level divided by total *FLT1* transcript level [154]. HsFlt-1-i13, the second most abundant placental *FLT1* transcript in preeclampsia, is generated by skipped splicing and extension of exon 13. Similarly, hsFlt-1-i14 is generated by skipped splicing and extension of exon 14. HsFlt-1-e15a and hsFlt-1-e15b contain alternatively spliced exons derived from intronic sequences (exon 15a and exon 15b, respectively). The most abundant placental transcript, hsFlt-1-e15a contains exon 15a, which is located within a primate-specific AluSeq retrotransposon. The graph was adapted with permission from figures in publications of Heydarian et al. [153] and Shibuya M. [243]. Permissions for reuse of these original figures were obtained from Elsevier Ltd. and from the Proceedings of the Japan Academy, Series B, respectively.

Among the several isoforms of sFlt-1, the primate-specific sFlt-1-e15a has the most abundant expression in the human placenta in preeclampsia [94,153–155]. It is important in this context that preeclampsia was primarily considered to be a “human disease,” since only a few cases presenting with preeclampsia-like symptoms have been reported among other primates (pregnant gorillas and chimpanzees), and preeclampsia has not been observed in any other species [156–159] (**Fig 1**). It is possible that this primate-specific sFlt-1-e15a isoform has an important role in the development of preeclampsia in humans and in some extent in the development of preeclampsia-like symptoms in other anthropoid primates, although the observations on non-human primate pregnancies are limited [160–162]. Another concept states that preeclampsia is the primary consequence of failure of deep trophoblast invasion, as humans have hemochorial placentation with uniquely deep trophoblast invasion that is in certain extent similar to chimpanzees and gorillas, which is not the case in other species [159,163,164].

Because of the anatomical and physiological uniqueness of human placentation, it has been impossible to develop adequate animal models to mimic the early placental stages of preeclampsia, especially for early-onset preeclampsia associated with severely impaired placental development. On the other hand, various animal models of preeclampsia could model the common terminal pathway of preeclampsia [165,166]. Most of these models either utilized hypertensive mouse strains [167,168] or were based on impaired uterine perfusion [169–180], nitric oxide synthase function [181–184], metabolic functions [185–187], oxidative and nitrosative stress [188,189], or altered renin-angiotensin system functions [190–193]. Other preeclampsia models generated systemic maternal inflammatory response [194,195] or utilized the overexpression of anti-angiogenic molecules [87,151,196–202]. Among the various species used in these studies, mice turned to be a good model animal for the study of late-onset preeclampsia, since they have hemochorial placentation similar to humans [203,204]. Although trophoblast invasion is limited in mice, placentation events can be somewhat comparable to those in humans [203–208].

Many previous models of preeclampsia in rodents had several technical constraints, which limited follow-up during pregnancy and postpartum, including: 1) lack of appropriate imaging techniques to determine pregnancy status in early gestation posed by the small size of these rodents; 2) lack of urine protein measurements due to difficulties with urine collection techniques; 3) limitations in continuous and/or non-stressed blood pressure monitoring; and 4) the lack of postpartum monitoring. 5) Moreover, previous anti-angiogenic models of preeclampsia in rodents extensively used a truncated sFlt-1(1–3) mutant, which is not expressed in any species. In this study, we aimed to develop a biologically more relevant anti-angiogenic mouse model of preeclampsia by overexpressing the most abundant human placental hsFlt-1-e15a isoform in preeclampsia in order to detect its presumed *in vivo* pathologic effects. This model was also aimed at utilizing non-stressed blood pressure monitoring during pregnancy and postpartum, and included high-frequency ultrasound imaging and molecular biological techniques to overcome earlier technical limitations.

## Materials and Methods

### Ethics statement

The study protocol (A#11-03-11) was approved by the Institutional Animal Care and Use Committee (IACUC) of Wayne State University (Detroit, MI, USA). Animal handling and care followed all standards in strict accordance with the recommendations in the “Guide for the Care and Use of Laboratory Animals” of the National Institutes of Health (NIH) [209]. All surgeries were performed under isoflurane anesthesia, and all efforts were made to minimize suffering. Mice were euthanized in accordance with the *“*Guidelines on Euthanasia” of the American Veterinary Medical Association, and the IACUC guidelines at Wayne State University.

### Animals and husbandry

Timed-pregnant CD-1 mice arrived from Charles River Laboratories (Wilmington, MA, USA) on gestational day (GD) 5, and then acclimated for two days before the experiments. Mice were kept separately in standard-size filter top rodent cages and fed with *ad libitum* water and food. Constant temperature (24±1°C) and humidity (50±5%) were maintained in the animal room with a daily regular 12:12 hour light-dark period. Mice were monitored daily for food and water intake, vital signs, activity, and behavior. Incision sites were examined daily to detect any signs of infection and/or inflammation, and genital regions for signs of vaginal discharge or preterm labor. Animals were excluded from the study in case of miscarriage or complications from surgery.

### Determination of pregnancy status with ultrasound

**Fig 2:**
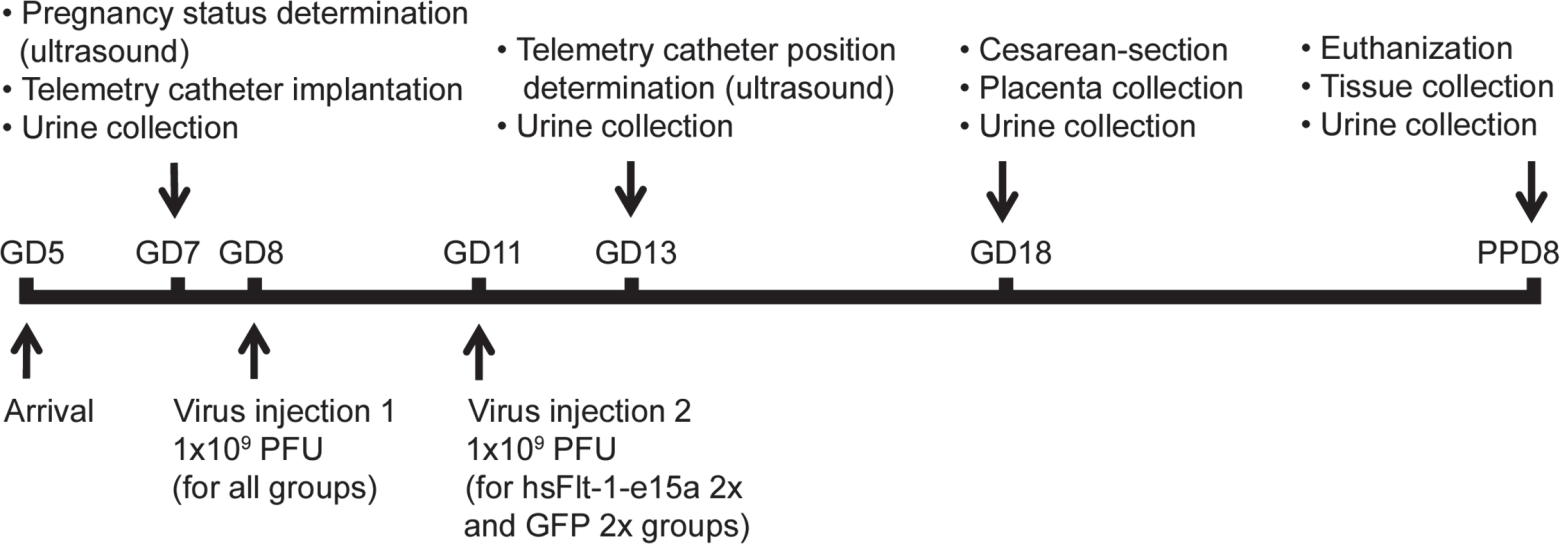
Experimental procedures. The flow-chart shows the experimental procedures performed at certain time-points during the study. GD, gestational day; PPD, postpartum day; PFU, plaque forming unit.

**Fig 3:**
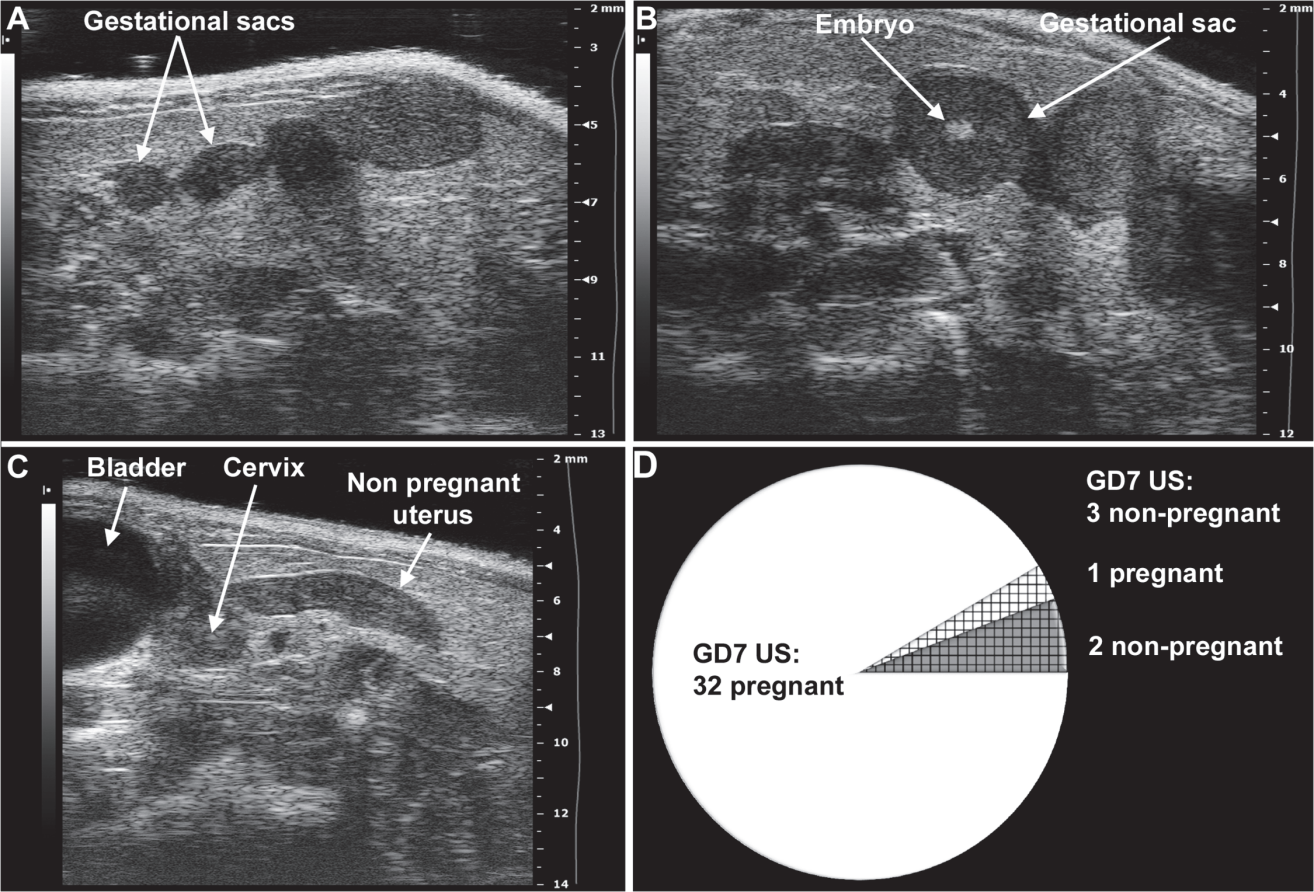
Determination of mouse pregnancy status with a 55MHz ultrasound probe. (A) Pregnant uterus on GD6. Gestational sacs of 1.8mm–2.7mm were observed in the proximity of the abdominal surface without visible signs of an embryo. (B) Pregnant uterus on GD7. Advanced endometrial reaction and the presence of an embryo were visible in the gestational sacs. (C) Non-pregnant uterus of a mouse seven days after mating, equivalent to GD7. (D) The pie chart shows that pregnancy could be diagnosed in 32 (white) of the 35 mice with high-frequency ultrasound on GD7. Of three mice diagnosed as non-pregnant on GD7 (shading), two were non-pregnant (grey shading), while one carried a pregnancy to term (white shading).

Timed-pregnant CD-1 mice arrived on GD5 (**Fig 2**), when the vendor guarantees only a 75% pregnancy rate. Ultrasound scans were performed on GD6 (n = 12) or GD7 (n = 35) to determine pregnancy status. Anesthesia was induced by inhalation of 4–5% isoflurane (Aerrane, Baxter Healthcare Corporation, Deerfield, IL, USA) and 1–2 L/min of oxygen in an induction chamber. Anesthesia was maintained with a mixture of 2% isoflurane and 1–1.5 L/min of oxygen. Expired gas from mice and leaking gas from the anesthesia mask were scavenged by a ventilation system connected to a charcoal filter canister. Mice were positioned on a heating pad and stabilized with adhesive tape, and then fur was shaved from the abdomen and neck. Body temperature was supported in the range of 37±1°C and detected with a rectal probe. Respiratory and heart rates were monitored throughout the ultrasound scans (Vevo Imaging Station, Visual Sonics Inc., Toronto, ON, Canada). After the 55MHz linear ultrasound probe (Vevo 2010, Visual Sonics Inc.) was fixed and mobilized with a mechanical holder, pregnancy status was evaluated while looking for signs of a gestational sac (GD6, **Fig 3A**), as well as an embryo and an advanced endometrial reaction (GD7, **Fig 3B**) [210–218].

### Implantation of the telemetric blood pressure monitoring system

**Fig 4:**
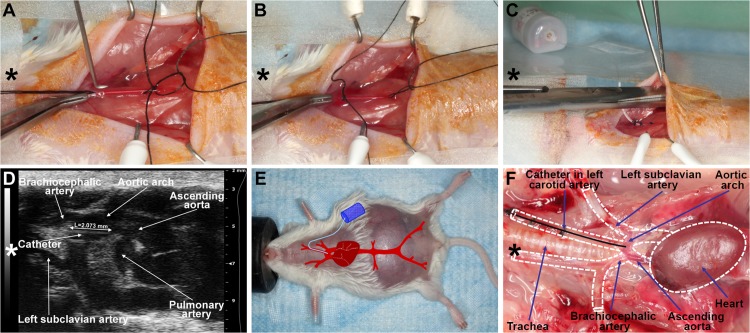
Implantation with a telemetric blood pressure monitoring system. (A) On GD8, after isolation and ligation of the left common carotid artery at the level of bifurcation, a small arteriotomy was prepared with a 25G tip needle, (B) and the blood pressure monitoring catheter was positioned into the aortic arch with the assistance of the vessel cannulation forceps. (C) The transmitter was placed into a subcutaneous pocket in the left flank and preformed with blind dissection. (D) On GD13, the position of the telemetry catheter was determined with a 55MHz ultrasound probe. The catheter tip is situated in the aortic arch, and the intra-aortic part of the catheter reaches the optimal 2mm length. (E) On GD18, a pregnant mouse is shown before a cesarean section. The incision line of the telemetry surgery healed completely. The projected graph illustrates the position of the intra-aortic catheter and the subcutaneous telemetric blood pressure transmitter. (F) On PPD8, the catheter position, aortic arch, and main arterial branches were visualized after autopsy in the mouse mediastinum and chest. The dotted lines show the heart and main arteries of the mediastinum. (A-F) Head orientations are shown with asterisks.

Mice with confirmed pregnancies underwent telemetric blood pressure monitoring system implantation on GD7. Isoflurane anesthesia was induced and maintained, and gas was scavenged as previously described. Mice were laid on their backs on the surgical platform, and their upper incisors and limbs were stabilized. Body temperature was controlled by a T/Pump warm-water circulating blanket (Gaymar Industries, Inc. Orchard Park, NY, USA). The incision site was scrubbed with Betadine (Purdue Pharma L.P., Stamford, CT, USA); and 2% lidocaine (0.5 mg/kg, Vedco Inc., St. Joseph, MO, USA) and 0.5% bupivacaine (1.5mg/kg, Hospira Inc., Lake Forest, IL, USA) were injected subcutaneously (s.c.) before the incision, following the rules of aseptic surgery [219]. An approximate 1.5cm midline incision was made on the neck, and the salivary glands were gently dissected and retracted laterally with elastic plastic stay hooks. An approximate space of 1cm of the left common carotid artery was exposed from the bifurcation in the direction of the heart, with the intention not to injure the vagal nerve. After carotid artery ligation at the level of bifurcation, arteriotomy and cannulation were prepared with the assistance of a 25G tip needle (**Fig 4A**), and the blood pressure monitoring catheter (TA11PA-C10 or HD-X11, Data Sciences International, St. Paul, MN, USA) was positioned into the aortic arch (**Fig 4B**). The catheter was fixed with 6/0 non-absorbable braided surgical silk sutures (Teleflex Medical, Coventry, CT, USA), and the transmitter was placed in a subcutaneous pocket in the left flank, preformed with blind dissection (**Fig 4C**). After repositioning the salivary glands over the catheter, the skin was closed with a continuous 6/0 non-absorbable monofilament polypropylene suture (CP Medical, Portland, OR, USA). Postoperative pain was reduced with s.c. injection of carprofen (5 mg/kg/24h, Rymadil, Pfizer Inc., New York, NY, USA), and with the administration of lidocaine and bupivacaine adjacent to the surgical incision site. In order to avoid post-surgical dehydration, 0.5ml of 0.9% saline solution was s.c. injected. During the postoperative period, mice were kept in their cages, with one-half of each cage placed on a warm-water circulating blanket, and vital signs were regularly checked.

### Determination of the telemetry catheter position with high-frequency ultrasound

Transmitter catheter tip positions were examined with the 55MHz linear ultrasound probe (Visual Sonics Inc.) during the routine GD13 ultrasound scans. The ultrasound probe was fixed and mobilized with a mechanical holder, and the transducer was moved downward toward the chest. The left carotid artery, aortic arch, and ascending aorta were visualized, and the position of the catheter tip was determined (**Fig 4D**).

### Adenoviral gene delivery

Recombinant adenoviruses expressing enhanced green fluorescent protein (GFP) or hsFlt-1-e15a (NP_001153502.1) under the control of a cytomegalovirus promoter (Ad-CMV-GFP and Ad-CMV-hsFlt-1-e15a, respectively) were constructed and titered by Vector BioLabs (Philadelphia, PA, USA). Mice were divided into four groups [hsFlt-1-e15a 1x (n = 6), hsFlt-1-e15a 2x (n = 5), GFP 1x (n = 4), and GFP 2x (n = 5)] according to the number of viral construct injections. All mice were injected with adenovirus constructs [1x10^9^ plaque-forming units (PFU) in 100μl] via the tail vein on GD8, and a subset of mice (GFP 2x and hsFlt-1-e15a 2x) was repeatedly injected with 1x10^9^ PFU adenoviral constructs on GD11. All of these mice underwent the subsequently described procedures.

### Telemetric blood pressure monitoring

As postoperative pain has a strong effect on blood pressure, telemetry monitoring was started on GD10, three days after the catheter implantations on conscious, unrestrained animals, and was continued until postpartum day (PPD) 7 using the Dataquest A.R.T. 4.31 acquisition and analysis system (Data Sciences International). Blood pressures were recorded for 10s every five minutes for at least 8–12 hours a day during both the light and dark cycles.

### Ultrasound-guided bladder puncture (cystocentesis)

Ultrasound-guided cystocentesis was performed on GD7, GD13, GD18, and PPD8 under isoflurane anesthesia. Urine samples were obtained using a micro-injection system and a linear 55 MHz high-frequency ultrasound probe (Visual Sonics Inc.), and stored at -80°C until analysis. After the procedure, the fluid loss was supplemented by subcutaneous injection of pre-warmed 0.9% NaCl (10–15 microliters/g/hour) into the midscapular region of the mice according to the recommendation of the IACUC and the Division of Laboratory Animal Resources (DLAR) of Wayne State University.

### Cesarean section

**Fig 5:**
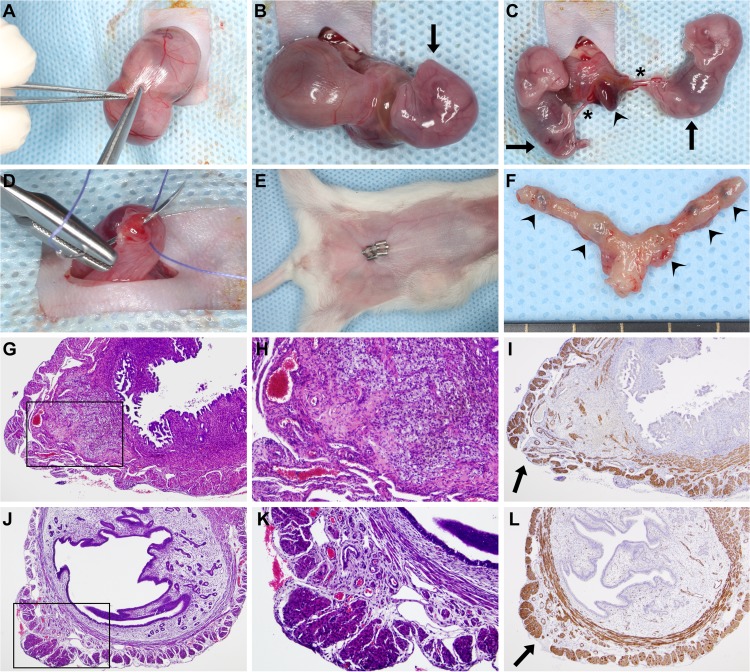
Survival cesarean section. (A) After a 1cm–1.5cm midline abdominal incision, a short segment of one uterine horn was exteriorized, and a 3mm–5mm longitudinal hysterectomy was performed on the opposite side of the mesometrial arterial arcade. (B-C) As the uterine wall could be easily dilated, this minimal incision enabled the delivery of two to three fetuses and their placentas using gentle fingertip pressure. An arrowhead depicts a placenta, stars depict umbilical cords, and arrows point to the fetuses. (D) Hysterectomies were closed with a single 4/0 absorbable multifilament suture. (E) After abdominal lavage with 0.9% sterile saline, the abdominal wall was closed with an absorbable multifilament continuous suture, and the skin was closed with 7mm-wide staples. The image shows a surgical field before euthanization on PPD8. (F) One uterus harvested after euthanization on PPD8. Arrowheads show hysterectomy sutures. (G) H&E staining of a uterine cross-section of a control mouse euthanized on PPD8 shows granulation tissue with enlarged vessels, foam cells, myofibroblasts, macrophages, and hemosiderin deposition. The image inside the black box is magnified in Subfigure H. (I) SMA immunostaining of the same uterus. The arrow depicts the cesarean section incision site with the disruption of the two-layered myometrium. (J) H&E staining of a uterine cross-section of a control mouse euthanized on PPD77 shows granulation tissue and the complete healing of the two-layered myometrium. The image inside the black box is magnified in Subfigure K. (L) SMA immunostaining of the same uterus. The arrow depicts a suture site. (G, I, J and L: 40x magnifications, H and K: 100x magnifications).

Mice underwent cesarean delivery on GD18. Pre- and intra-operative preparation of the mice (i.e., isoflurane anesthesia induction and maintenance, eye protection, surgical stabilization and body temperature control, skin disinfection, and local analgesia) were performed as in the case of the telemetry device implantation. After a short (1–1.5cm) midline abdominal incision in the area where fur had been previously removed, a short segment of one uterine horn was exteriorized at once, and kept moisturized with sterile 0.9% saline. According to the number of pups, two to three exteriorizations and minimal (3–5mm) longitudinal midline hysterectomies were made on each horn, on the opposite side of the mesometrial arterial arcade, while keeping the residual parts of the uterine horn inside the abdominal cavity to avoid contamination (**Fig 5A**). After delivering pups and placentas (**Fig 5B and 5C**), minimal incisions were closed with a single 4/0 absorbable multifilament polyglycolic acid suture (CP Medical, Portland, OR, USA) (**Fig 5D**). Then, lavage was applied to the abdominal cavity with 2–3ml of 0.9% sterile saline. The abdominal wall was closed with a continuous 4/0 absorbable multifilament polyglycolic acid suture (CP Medical), and the skin was closed with 7mm staples (Braintree Scientific Inc., Braintree, MA, USA) (**Fig 5E**). Body fluids were replenished by an injection of 0.5ml of 0.9% sterile saline subcutaneously. Postoperative pain was reduced with s.c. carprofen (Pfizer Inc.), as well as with the injection of lidocaine and bupivacaine adjacent to the incision line. Postoperative care was similar to that which followed the telemetry system implantation.

### Tissue collection

Following cesarean section on GD18, each fetus was separated from the placenta and umbilical cord. All fetuses and placentas were weighed with a Scout Pro SP402 digital scale (Ohaus Corp., Pine Brook, NJ, USA). The first placentas adjacent to the uterine cervix in both uterine horns were fixed in 4% paraformaldehyde (PFA) diluted with phosphate buffered saline (PBS, Gibco, Life Technologies Corporation, Grand Island, NY, USA) for 24h, then dehydrated in 70% graded ethanol (Richard-Allan Scientific Dehydrant, Thermo Fisher Scientific Inc., Waltham, MA, USA), and embedded in paraffin for histopathological examinations. The second placentas adjacent to the uterine cervix were collected and homogenized in TRIzol (Invitrogen, Life Technologies Corporation, Carlsbad, CA, USA) and stored at -80°C until gene expression analyses.

After the euthanization of dams on PPD8, tissues from several organs (spleen, uterus, liver, kidney, and brain) were dissected and sectioned. Tissues were fixed in 4% PFA for 24h, then dehydrated in 70% graded ethanol, and embedded in paraffin for histopathological examinations. Tissues were also homogenized in TRIzol reagent and stored at -80°C until gene expression analyses. To evaluate the changes in uterine histology with time after the cesarean sections, additional untreated mice were euthanized on PPD38 (n = 3), PPD50 (n = 3), and PPD77 (n = 3), respectively, and uteri were processed as those on PPD8.

### Total RNA isolation, cDNA generation, and quantitative real-time RT-PCR

Tissues were homogenized in TRIzol reagent with a homogenizer (Pro Scientific Inc., Oxford, CT, USA) immediately after tissue collection. Total RNAs were isolated using the QIAshredder (Qiagen, Valencia, CA, USA) and the RNeasy Mini Kit (Qiagen), according to the manufacturer’s instructions. Complementary DNAs were generated with SuperScript III First-Strand Synthesis System (Invitrogen). Quantitative real-time RT-PCR assays were performed on the Biomark System (Fluidigm, San Francisco, CA, USA) using TaqMan assays (Applied Biosystems, Life Technologies Corporation, Foster City, CA, USA) for human *FLT1* (Hs01052961_m1), *GFP* (Mr04097229_mr), and the mouse endogenous control gene *Gapdh* (Mm99999915_g1) according to the manufacturer’s recommendation.

### Histopathological evaluation of tissues

Five-μm-thick sections of paraffin embedded placenta, kidney, and uterus tissue blocks were serially cut, mounted on silanized slides, deparaffinized, and rehydrated in descending grades of ethanol. Selected levels of all tissues were then stained with hematoxylin and eosin (H&E) to evaluate general morphology, and selected levels of all kidneys were stained with periodic acid Schiff (PAS) reagent for the visualization of basement membranes of glomerular capillary loops and tubular epithelium. Histopathological examination of these tissue sections was performed on an Olympus BX50F light microscope (Olympus America Inc., Melville, NY, USA) by a pathologist (FQ). Kidney sections were evaluated for glomerular endotheliosis (e.g. ballooning of tips of capillary loops, capillary endothelial swelling, occlusion of glomerular capillaries) in at least 20 glomeruli in the inner cortex of one kidney in each animal.

### Immunohistochemistry

Selected layers of uteri were immunostained for CD68 and smooth muscle actin (SMA). Immunostainings were performed using a rabbit anti-mouse SMA polyclonal antibody (1:300 dilution; Abcam Inc., Cambridge, MA, USA) and the Bond Polymer Refine Detection Kit (Leica Microsystems, Wetzlar, Germany) on a Leica Bond Max automatic staining system (Leica Microsystems), or using a rabbit anti-mouse CD68 polyclonal antibody (1:150 dilution; Abcam Inc.) and the DAB Map Detection Kit (Ventana Medical Systems, Inc., Tucson, AZ, USA) on a Ventana automatic staining system (Ventana Medical Systems, Inc.).

### Aortic ring assays

Aortic ring assays were performed as previously described [220,221]. Briefly, thoracic aortas were dissected from euthanized mice and placed in a Petri dish containing DMEM+GlutaMAX low glucose medium (Gibco, Life Technologies Corporation). The peri-adventitial fibro-adipose tissue was removed, then aortas were sectioned into 1mm-long rings, and incubated in 12-well plates at 37°C in Opti-MEM+GlutaMAX reduced serum medium (Gibco, Life Technologies Corporation) overnight for serum starvation. The serum-starved aortic rings were then placed into 96-well tissue culture plates pre-coated with 50μL of Growth Factor Reduced BD Matrigel Matrix (BD Biosciences, Bedford, MA, USA). Then, aortic rings were covered with an additional 50μL of Matrigel and 100μL of Opti-MEM medium supplemented with 1% Penicillin–Streptomycin (Gibco, Life Technologies Corporation), 2.5% fetal bovine serum (FBS; Atlanta Biologicals, Lawrenceville, GA, USA), and 30ng/mL of vascular endothelial growth factor (VEGF-A; ProSpec, East Brunswick, NJ, USA). Plates were incubated at 37°C for six days with a change of medium every second day. After six days of incubation, aortic rings were fixed with 4% PFA diluted with PBS (Gibco, Life Technologies Corporation), and images were obtained with an Olympus 1X51 inverted microscope equipped with an Olympus DP25 digital camera (Olympus, Tokyo, Japan).

### Albumin-creatinine immunoassays

Urine specimens collected on GD6/GD7, GD13, GD18 and PPD8 were examined for murine urinary albumin with the Albuwell kit (Exocell Inc., Philadelphia, PA, USA), and for creatinine with the Creatinine Companion assay employing the alkaline picrate method (Exocell Inc.).

### Adenoviral infection of BeWo cells

BeWo cells (American Type Culture Collection, Manassas, VA, USA) were cultured with F12 medium supplemented with 10% FBS and 1% Penicillin/Streptomycin (all from Life Technologies Corporation). Cells were plated on either 6-well plates (0.5x10^6^/well) or 35mm cell culture dishes, and infected with Ad-CMV-GFP or Ad-CMV-hsFlt-1-e15a at a multiplicity of infection of 100. After 16h of infection, cell supernatants were removed, and BeWo cells were washed with PBS and used for Western blotting or confocal imaging.

### Protein isolation and Western blot

Total protein from BeWo cell samples was extracted with the RIPA lysis buffer (Sigma-Aldrich Co., St Louis, MO, USA) containing Complete Mini Protease Inhibitor Cocktail Tablets (Roche, Indianapolis, IN, USA). Protein concentrations were determined with the Quick Start Bradford Protein Assay (Bio-Rad, Hercules, CA, USA). Twenty micrograms of total protein from each sample were electrophoresed on 4–12% SDS-PAGE gels (Life Technologies Corporation), and electro-transferred onto nitrocellulose membranes (Bio-Rad). Membranes were probed with goat anti-human Flt-1 polyclonal antibody (AF321, R&D Systems, Inc., Minneapolis, MN, USA) in a 1:2,000 dilution at 4C° for 16h, and then with peroxidase-conjugated anti-goat IgG (Vector Laboratories, Burlingame, CA, USA) in a 1:5,000 dilution at room temperature for 1h. Protein bands were developed using the ChemiGlow Western Blotting Detection Reagents (Protein Simple, Santa Clara, CA, USA), and then scanned and imaged with a Fujifilm LAS-4000 Image Reader (GE Healthcare, Piscataway, NJ, USA).

### Confocal microscopy

BeWo cells infected with adenovirus constructs and cultured in 35mm cell culture dishes were mounted with ProLong Gold Antifade Reagent and 4',6-diamidino-2-phenylindole (DAPI; Invitrogen) followed by confocal microscope imaging using a Leica TCS SP5 spectral confocal system (Leica Microsystems CMS GmbH, Mannheim, Germany). Confocal imaging was performed at the Microscopy, Imaging and Cytometry Resources Core of the Wayne State University School of Medicine.

### Data and statistical analyses

#### Blood pressures

Mean arterial pressures (MAP) were calculated from systolic and diastolic blood pressures for each time point and animal, and then were averaged. The mean MAP values on GD10 were subtracted from all MAP values to obtain normalized ΔMAP values. We fitted the MAP and the ΔMAP data with a Linear Mixed Effects Models (LME) [222] for the time intervals before and after cesarean delivery, respectively. These models included explanatory variables such as the treatment (GFP or hsFlt-1-e15a) or the dose (1x or 2x), and a continuous measure of time (gestational day or postpartum day) while allowing a random intercept for each animal. An interaction was allowed between the treatment and time, and therefore, we could test if the slope of the MAP or ΔMAP over time was different between the treatments. We relaxed the linear fixed effect patterns to quadratic for the analysis of time intervals after cesarean delivery. Since blood pressure has a circadian daily rhythm in mice, we also examined blood pressures in 12-hour light and dark cycles separately.

#### Urine albumin/creatinine ratios

Albumin/creatinine ratios between the hsFlt-1-e15a and GFP groups on different time points were compared with the Student's t-test.

#### Gene expression profiling

Relative gene expressions were quantified by averaging target (*FLT1* or *GFP*) and reference (*Gapdh*) gene Ct values over technical replicates, and then by subtracting mean target gene Ct values from mean reference gene Ct values within the same sample. The Student's t-test was used to compare gene expression levels between treatments in a given tissue. To examine the dose effect on gene expression for each tissue, we computed the percentage of samples expressing a given gene when over-expressed with a given dose of that gene. Statistical comparison on the percentages across all tissues between the two doses was performed with the one-tailed paired Student's t-test.

#### Aortic ring assays

The angiogenic response of the aortic rings was analyzed by quantifying the microvessel outgrowth. A ruleset was developed using Definiens Developer XD2 (Definiens, Munich, Germany) to analyze the transmitted light images. A new image layer (or channel) with enhanced local contrast was produced using “contrast to neighbor pixels” to distinguish the newly formed microvessels sprouting from the aortic ring. Next, a series of segmentation and classification operations was performed on the channel to exclude the ring from the area measurements, and the total area of the objects determined to be “outgrowth” was reported. Data were averaged on the picture level for the same ring, then further averaged on the ring level for the same animal, followed by a Student's t-test for group comparisons.

#### Fetal survival rates, fetal and placental weights

The fetal survival rate (number of live fetuses / number of total fetuses) for each mouse was computed, and the non-parametric Kruskal-Wallis test was used for multiple group comparisons. Fetal weights, placental weights, and placental/fetal weight ratios were compared with the two-way ANOVA test and with a linear mixed effects model.

## Results

### Pregnancy status determination with high-resolution ultrasound

Ultrasound examinations were performed with a 55MHz linear probe to corroborate pregnancy before the implantation of a telemetry catheter. On GD6, only 1.9mm-2.7mm gestational sacs could be visualized (**Fig 3A**). Since an advanced endometrial reaction and the embryo were already visible on GD7, we later decided to perform the ultrasound examinations on GD7 (**Fig 3B**). In non-pregnant animals, none of these signs were visible (**Fig 3C**). In the set of 35 mice scanned on GD7, pregnancy was diagnosed in 32 mice (**Fig 3D**). Among the three mice diagnosed as non-pregnant, two were non-pregnant, and one delivered at term.

### Implantation and evaluation of telemetry devices

Telemetry catheter implantations took place only after pregnancy was confirmed with ultrasound, according to the guidelines provided by Data Sciences International (**Fig 4**). The average length of the implantation surgeries varied between 20–35 minutes, with the TA11PA-C10 device requiring a shorter duration of surgery than the HD-X11 transmitter. Ultrasound examinations on GD13 showed that all animals had a correctly positioned telemetry catheter. The rate of uncomplicated telemetry system implantations was 79% (30/38). According to the type of device implanted, 86% (18/21) were uncomplicated telemetry implantations in cases using the TA11PA-C10 transmitter, and 71% (12/17) in cases using the HD-X11 device. Among these eight cases with complications, three mice implanted with the TA11PA-C10 devices and one mouse implanted with the HD-X11 device had abnormal body posture and seizures, suggesting ischemic brain damage, while four mice implanted with the HD-X11 devices presented with transmitter body exteriorization.

### Survival cesarean surgery

We developed a new survival cesarean surgery, in which only a short segment of one uterine horn is exteriorized at a time. In total, two or three short longitudinal hysterectomies were performed on each horn, in which pups and placentas could easily be delivered with a gentle push (**Fig 5A–5F**). Due to the minimal invasiveness of this aseptic technique, administration of appropriate pain medication, and replenishment of lost body fluids, the eight-day survival rate of this new surgery was 100% (30/30) compared to the 85% reported in the literature [223]. Histopathological examination of H&E-stained and SMA-immunostained uterine cross-sections showed granulation tissues with enlarged vessels, foam cells, myofibroblasts, macrophages, and hemosiderin deposits on PPD8 (**Fig 5G–5I**). The endometrium was completely healed on PPD77 in all examined cases, and the two-layered myometrium showed complete healing of the inner layer and focal disruption at suture sites in the outer layer. Suture granuloma was observed in the submucosa at the incision site (**Fig 5J–5L**).

### Overexpression of human placental sFlt-1-e15a isoform and GFP with adenoviral vectors

**Fig 6:**
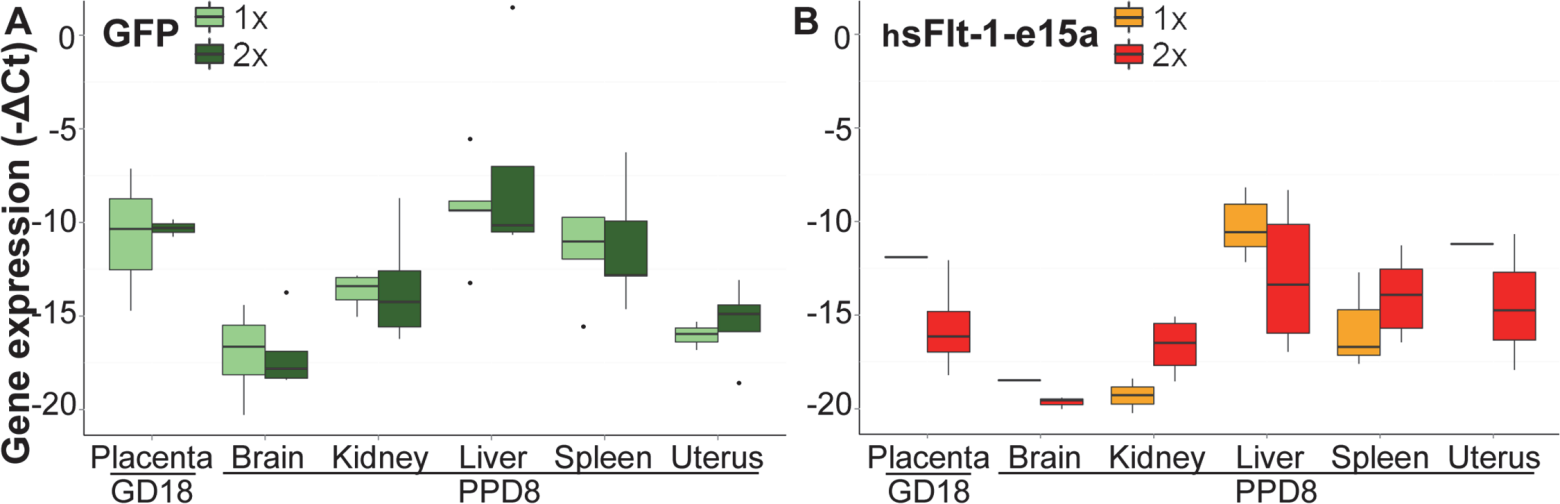
Profiling of hsFlt-1-e15a and GFP expression. Boxplots show the expression profile of GFP (A) or hsFlt-1-e15a (B) mRNAs in placentas harvested on GD18 and in five tissues harvested on PPD8. Adenovirus doses (1x10^9^ or 2x10^9^ plaque-forming units) are depicted with different colors. Expression of hsFlt-1-e15a and GFP mRNAs was highest in the liver. Viral dose-effect was not seen in GFP expression; however, there was a dose-effect in hsFlt-1-e15a expression. The number of hsFlt-1-e15a expressing placenta, brain and uterine tissues was low in the hsFlt-1-e15a 1x group.

To compare the expression pattern of hsFlt-1-e15a and GFP mRNAs, total RNAs were isolated from placentas harvested on GD18, as well as from brain, kidney, liver, spleen, and uterus tissues harvested on PPD8. According to the qRT-PCR analysis, hsFlt-1-e15a and GFP mRNA expression was highest in the liver (**Fig 6A and 6B**). The placental expression of hsFlt-1-e15a and GFP was not directly comparable with their expression in the five investigated maternal tissues because of the difference in tissue sampling time points (GD18 vs. PPD8). Viral dose-effect (1x10^9^ PFU vs. 2x10^9^ PFU) was not seen in GFP expression, since there was no difference between the percentage of tissues expressing GFP in the two control groups (GFP 1x: 80.0% vs. GFP 2x: 83.3%; p = 3.5x10^-1^). On the contrary, there was a dose-effect in the expression of hsFlt-1-e15a, which had lower expression in all investigated tissues than GFP. For mice in the hsFlt-1-e15a 1x group (1x10^9^ PFU), the average rate of tissue samples with detectable hsFlt-1-e15a expression was 46.7%, and 72.2% in the hsFlt-1-e15a 2x (2x10^9^ PFU) group (p = 9.7x10^-3^). The hsFlt-1-e15a mRNA expression was 6.3-fold higher in the kidneys of hsFlt-1-e15a 2x mice than of hsFlt-1-e15a 1x mice (p = 5.3x10^-2^). The number of hsFlt-1-e15a expressing placenta, brain, and uterine tissues was low in the hsFlt-1-e15a 1x group.

To confirm the placental overexpression of hsFlt-1-e15a and GFP proteins in the trophoblast *in vitro*, we infected BeWo human trophoblast-like cells with Ad-CMV-GFP or Ad-CMV-hsFlt-1-e15a, and used a human anti-Flt-1 antibody for Western blotting or confocal microscopy for GFP signal detection. Western blotting revealed that non-infected BeWo cells expressed low amounts of the 185kDa Flt-1 membrane receptor as well as 145kDa and 110kDa sFlt-1 variants (**S1A Fig**). Ad-CMV-GFP-infected BeWo cells expressed these proteins to the same extent. Ad-CMV-hsFlt-1-e15a-infected BeWo cells overexpressed the 145kDa and 110kDa sFlt-1 variants. Confocal microscopy revealed cytoplasmic GFP expression in BeWo cells infected with Ad-CMV-GFP (**S1B Fig**), indicating also the efficient transfection of these trophoblastic cells with the adenoviral constructs.

### Blood pressure telemetry monitoring

**Fig 7:**
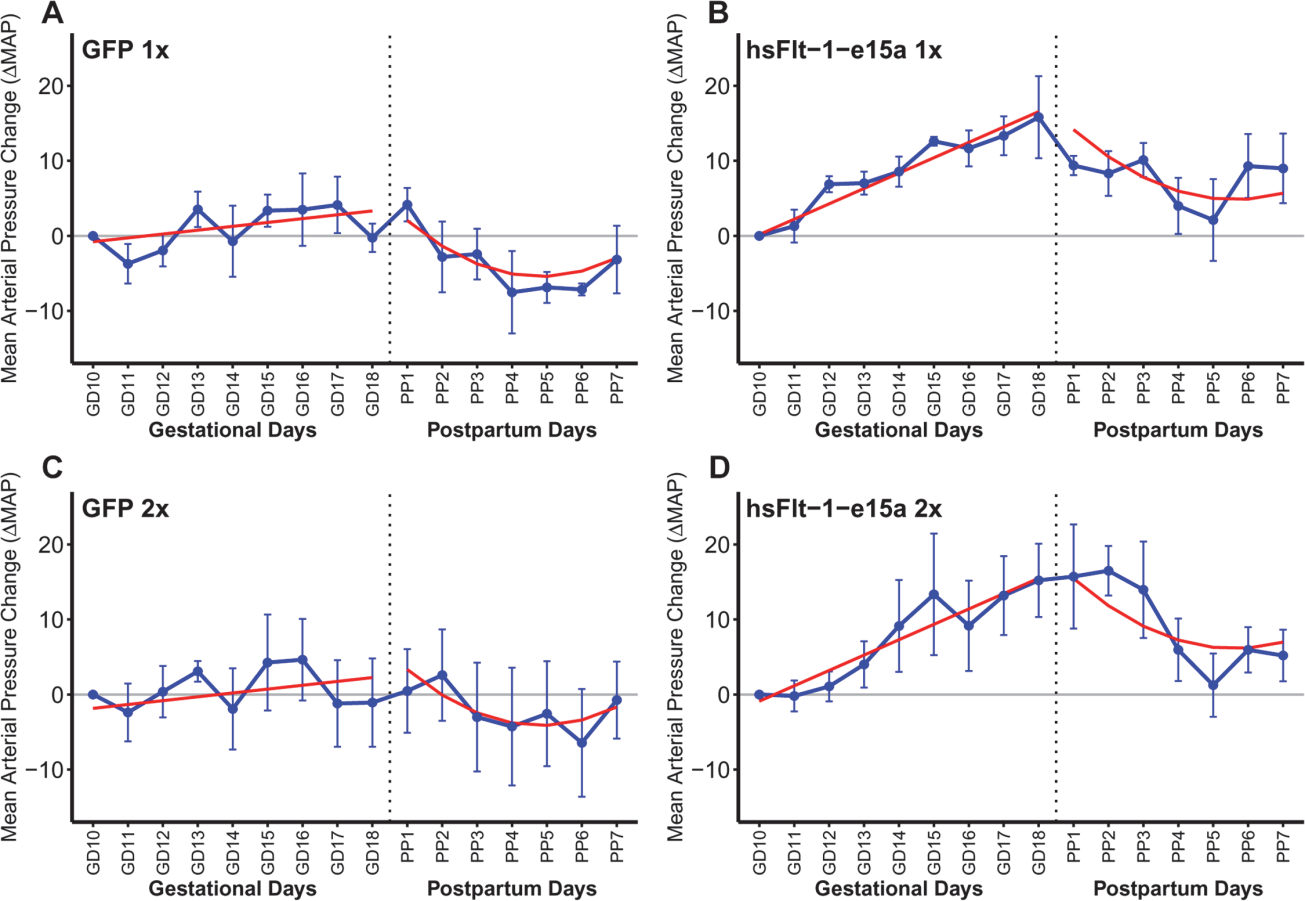
Blood pressure monitoring. X-axes show gestational days (GDs) and postpartum days (PPDs). Mean arterial pressure changes (ΔMAP) are depicted on the Y-axes. Blue dots represent ΔMAP values for given time-points, blue error bars show +/-standard errors. Red lines depict the ΔMAP patterns, fitted from the linear mixed effects models. During pregnancy (left sides of the sub-figures), there was no significant blood pressure elevation in control mice (A,C; ΔMAP slope = 0.513 mmHg/day; p = 0.187), whereas hsFlt-1-e15a treatment significantly increased blood pressure (B,D; ΔMAP slope = 2.05 mmHg/day; p = 8.09x10^-8^). The ΔMAP slope in the hsFlt-1-e15a group was higher compared to the controls (1.53 mmHg/day; p = 0.0043). The ΔMAP at parturition (GD18) was 13.2 mmHg higher in hsFlt-1-e15a-treated mice than in the control mice (p = 0.00107). After cesarean delivery (right sides of sub-figures), a similar quadratic pattern of ΔMAP was found in both control and hsFlt-1-e15a-treated mice, but ΔMAP dropped below the baseline in control mice (-1.96 mmHg; p = 0.560), while it remained above this in hsFlt-1-e15a treated mice (6.88 mmHg; p = 0.0346). There was no dose effect of the number of viral construct injections (1x10^9^ PFU vs. 2x10^9^ PFU) in hsFlt-1-e15a-treated or in control mice either before delivery (dose effect: -1.06 mmHg, p = 0.693) or in the postpartum period (dose effect: 1.30 mmHg, p = 0.763).

As our preeclampsia model allowed for postpartum monitoring, blood pressure measurements continued until PPD7. Blood pressures increased until cesarean delivery and then declined until tissue harvest in hsFlt-1-e15a overexpressing mice. Therefore, we fitted the data separately for the time periods before and after cesarean delivery. This analysis revealed that, prior to parturition, there was no significant change in the blood pressure over time in control mice (ΔMAP slope = 0.513 mmHg/day; p = 0.187) (**Fig 7A and 7C**). However, in response to hsFlt-1-e15a treatment, blood pressures increased over time (ΔMAP slope = 2.05 mmHg/day; p = 8.09x10^-8^). The ΔMAP slope in the hsFlt-1-e15a group was significantly higher compared to the controls (1.53 mmHg/day; p = 0.0043), resembling late-onset preeclampsia in humans (**Fig 7B and 7D**). The ΔMAP at parturition (GD18) was 13.2 mmHg higher in hsFlt-1-e15a-treated mice than in the control mice (p = 0.00107). Interestingly, a similar quadratic pattern of ΔMAP was found in both control and hsFlt-1-e15a-treated mice after cesarean delivery, suggesting a common pattern of drop of blood pressures. However, ΔMAP dropped below the baseline in control mice, while it remained above this in the postpartum period in hsFlt-1-e15a-treated mice. Indeed, ΔMAP on PPD7 was 1.96 mmHg below the baseline in control mice (p = 0.560), while it was 6.88 mmHg above the baseline in hsFlt-1-e15a-treated mice (p = 0.0346) (**Fig 7A–7D**).

We did not observe any effect of the number of viral construct injections (1x10^9^ PFU vs. 2x10^9^ PFU) in hsFlt-1-e15a-treated or in control mice either before delivery (dose effect: -1.06 mmHg, p = 0.693) or in the postpartum period (dose effect: 1.30 mmHg, p = 0.763).

When examining the day and night cycles separately, we observed that overall the ΔMAP was 2.67 mmHg higher during the night cycles than during the day cycles (p = 2.5x10^-3^) before cesarean delivery, and ΔMAP was 4.37 mmHg higher during the night cycles than during the day cycles (p = 2.7x10^-6^) after cesarean delivery (**S2 Fig**).

In addition to normalizing mean arterial blood pressures to obtain ΔMAPs, we also repeated the above analysis with mean arterial pressures (MAPs). As expected, the conclusions remained the same with regard to the trend before and after parturition for control and hsFlt-1-e15a-treated mice. Prior to parturition, there was no change in the blood pressures over time in control mice (MAP slope = 0.426 mmHg/day; p = 0.174) (**S3A and S3C Fig**). However, in response to hsFlt-1-e15a treatment, blood pressures increased over time (MAP slope = 2.02, mmHg/day; p = 1.13x10^-10^). The difference in the MAP slope in the hsFlt-1-e15a groups was higher compared to the controls (1.59 mmHg/day; p = 2.6x10^-4^) (**S3B and S3D Fig**). The only noticeable change was in the estimated effect of hsFlt-1-e15a treatment on GD18, which dropped from 13.2 mmHg in ΔMAP to 6.90 mmHg (p = 0.0339) in MAP. This difference could be attributed to the base-line group differences in MAP levels on GD10.

Similarly as above, we did not observe any effect of the number of viral construct injections (1x10^9^ PFU vs. 2x10^9^ PFU) in hsFlt-1-e15a-treated or in control mice either before delivery (dose effect: -3.95 mmHg, p = 0.119) or in the postpartum period (dose effect: -1.70 mmHg, p = 0.543).

### Evaluation of endothelial and kidney functions

**Fig 8:**
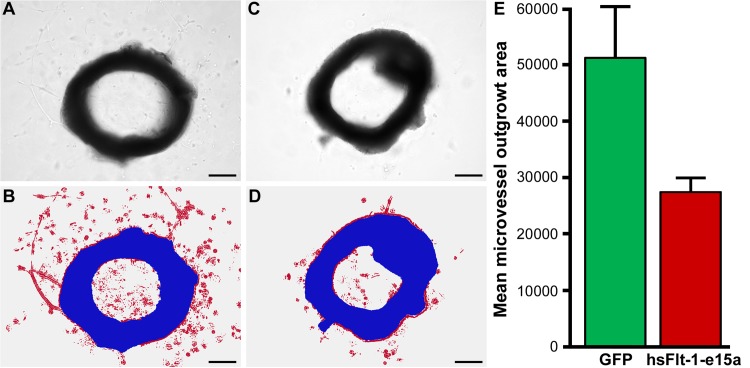
Aortic ring assays. (A) A light image of an aortic ring from a mouse injected with Ad-CMV-GFP. (B) An image of the same aortic ring, illustrating segmentation and classification, with the aortic ring shown in blue and the red color depicting the microvessel outgrowth area. (C) A light image of an aortic ring from a mouse injected with Ad-CMV-hsFlt-1-e15a. (D) An image of the same aortic ring, illustrating segmentation and classification, with the aortic ring shown in blue and the red color depicting the microvessel outgrowth area. (A-D) Scale bars depict 500μm. (E) The bar chart depicts the differences in mean microvessel outgrowth areas (pixels) between the GFP and hsFlt-1-e15a groups.

An aortic ring assay was utilized to investigate the *in vitro* endothelial functions of aortas collected postpartum. Aortic rings were analyzed after treatment with VEGF-A for six days by light microscopic imaging for the microvessel outgrowth area. We found that the mean microvessel outgrowth area was 46% smaller in hsFlt-1-e15a overexpressing mice than in controls (p = 0.012) (**Fig 8**).

**Fig 9:**
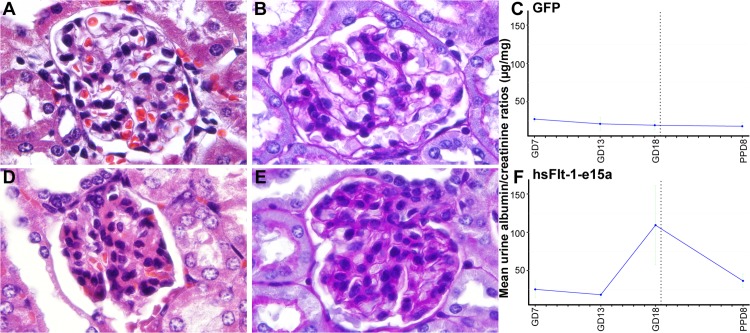
Histopathological and functional evaluation of the kidneys. Representative H&E (A) and PAS (B) stained sections show a morphologically normal glomerulus in a control animal. Representative H&E (D) and PAS (E) stained sections show a glomerulus with signs of swollen capillary endothelial cells and occlusion of glomerular capillaries in a mouse overexpressing hsFlt-1-e15a. (C,F) Charts depict albumin/creatinine ratios in urine specimens collected on gestational day (GD)7, GD13, GD18, and PPD8 from mice in the GFP (C) and hsFlt-1-e15a (F) groups. Mean urine albumin/creatinine ratios did not change in control mice; however, these increased by gestational days and then dropped postpartum in hsFlt-1-e15a overexpressing mice. Mean urine albumin/creatinine ratio on GD18 was higher in hsFlt-1-e15a-treated than in control mice (p = 4.4x10^-2^). The mean urine albumin/creatinine ratio on PPD8 was marginally significantly higher in hsFlt-1-e15a-treated (36.6±9.0μg/mg) than in control mice (18.0±4.9μg/mg; p = 0.06).

Histopathological examinations of the kidneys showed focal glomerular changes, including swollen capillary endothelial cells and occlusion of glomerular capillaries in mice overexpressing hsFlt-1-e15a (**Fig 9**). In addition, rare glomeruli appeared sclerotic. In contrast, there were no significant morphological changes in the glomeruli of control mice.

As *in vivo* functional evidence of kidney damage, we also determined albumin/creatinine ratios in urine samples obtained at GD6/GD7, GD13, GD18 and PPD8. Mean urine albumin/creatinine ratios did not change in control mice; however, these increased by GD18 and then dropped postpartum in hsFlt-1-e15a overexpressing mice. Mean (±2SE) urine albumin/creatinine ratio on GD18 was five times higher in hsFlt-1-e15a-treated (109.3±51.7μg/mg) than in control mice (19.3±5.6μg/mg; p = 4.4x10^-2^). The mean urine albumin/creatinine ratio on PPD8 was still marginally significantly higher in hsFlt-1-e15a-treated (36.6±9.0μg/mg) than in control mice (18.0±4.9μg/mg; p = 0.06) (**Fig 9**).

### Evaluation of fetal survival rate, placental and fetal weights

Fetal survival rate was not affected by hsFlt-1-e15a treatment, as no differences (p = 0.38) were found in the groups (GFP 1x: 100%; hsFlt-1-e15a 1x: 100%; GFP 2x: 96.43%; hsFlt-1-e15a 2x: 100%). Also in agreement with most studies, fetal weight (FW), placental weight (PW), and placental/fetal weight ratios (PFR) were not affected by hsFlt-1-e15a treatment (**S4 Fig**). Mean (±2SE) PWs, FWs, and PFRs were as follows: GFP 1x (PW: 0.109±0.009g; FW: 1.03±0.097g; PFR: 0.107±0.014g), hsFlt-1-e15a 1x (PW: 0.117±0.015g; FW: 1.08±0.059g; PFR: 0.109±0.017g), GFP 2x (PW: 0.098±0.014g; FW: 1.07±0.064g; PFR: 0.092±0.009g), and hsFlt-1-e15a 2x (PW: 0.102±0.010g; FW: 1.00±0.133g; PFR: 0.102±0.008g).

### Evaluation of a mouse with early-onset preeclampsia-like symptoms

**Fig 10:**
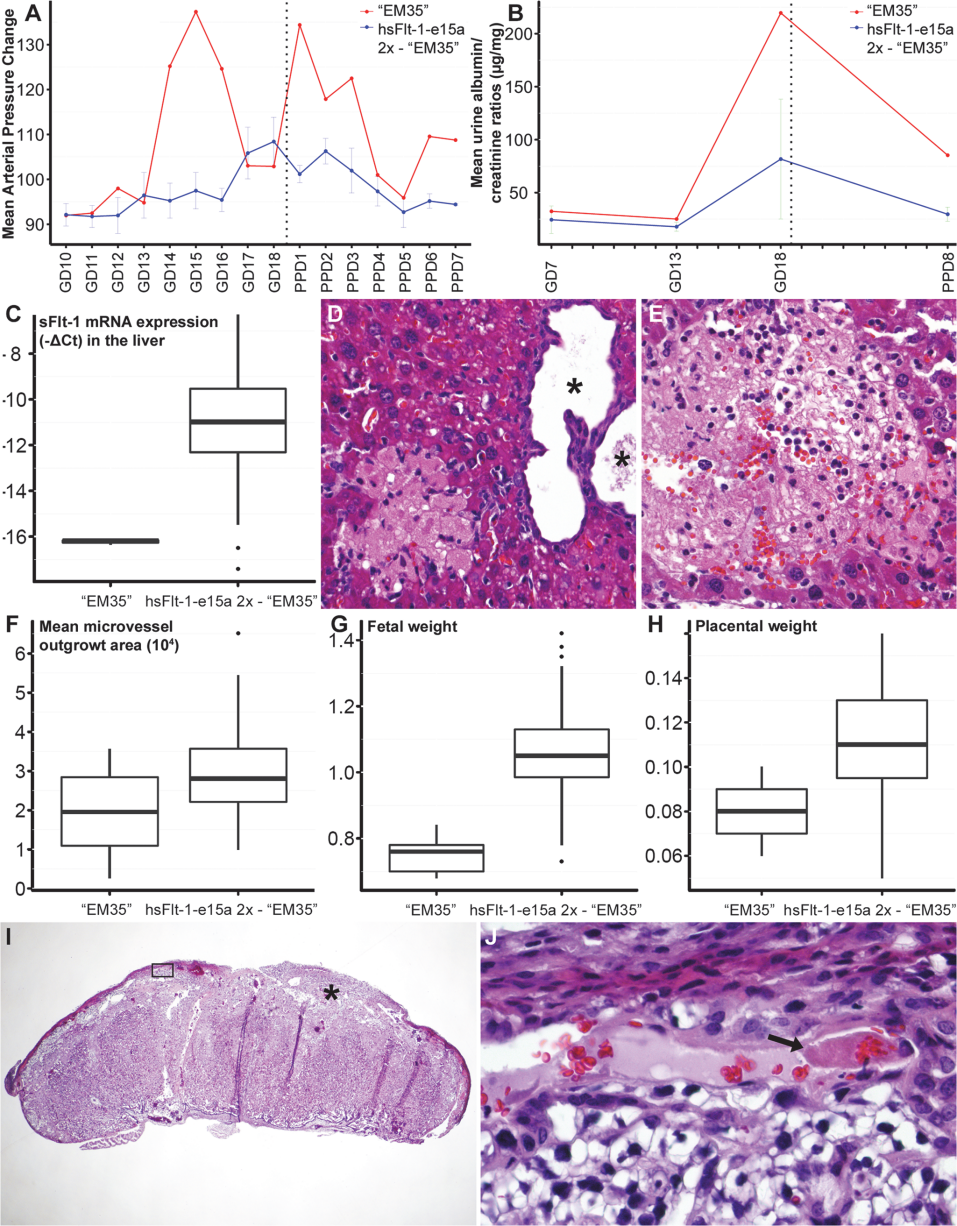
“EM35” mouse had early-onset preeclampsia-like symptoms associated with fetal growth restriction. (A) “EM35” mouse had a higher and earlier blood pressure peak than other hsFlt-1-e15a-treated mice, followed by the normalization of blood pressures and a second blood pressure peak on PPD1. (B) The urinary albumin/creatinine ratio on GD18 was higher in “EM35” mouse than in other hsFlt-1-e15a-treated mice. (C) HsFlt-1-e15a expression in the liver was lower in “EM35” mouse than in other hsFlt-1-e15a-treated mice. (D-E) Histopathological examination of the liver found a multiple cystic biliary hyperplasia in the liver (* in D), and multiple recent (D) and remote (E) infarcts in the parenchyma of liver. H&E staining, 200x magnifications. (F) The endothelial outgrowth area was the lowest in “EM35” mouse among hsFlt-1-e15a-treated mice. (G-H) Mean fetal (G) weights (0.749±0.029) and placental (H) weights (0.080±0.006) in “EM35” mouse were significantly lower than in other hsFlt-1-e15a-treated mice (fetal weight: 1.06±0.023, p = 2.017x10^-15^, placental weight: 0.111±0.004, p = 2.05x10^-6^). (I) Histopathological examination of a placenta from “EM35” mouse showed multiple thrombi in maternal decidual vessels (star and black box). H&E staining, 20x magnification. The image inside the black box is magnified to 800x in Subfigure I. (J) The arrow shows one of the thrombi.

It is noteworthy that one mouse in the hsFlt-1-e15a 2x group showed a different biological response despite receiving the same treatment (dosage and timing) as others in the group. The “EM35” mouse had a markedly higher and earlier (GD15) blood pressure peak than other hsFlt-1-e15a-treated mice (**Fig 10A**). This was followed by the normalization of blood pressures, and by a second blood pressure peak on PPD1. To investigate whether the extreme blood pressure elevation in this mouse could bias the effects estimated for hsFlt-1-e15a-treated mice, we re-analyzed ΔMAPs in the hsFlt-1-e15a group with or without including "EM35" mouse. This re-analysis showed that in the period before delivery the ΔMAP slope was 2.05 (p = 8.09x10^-8^) before and 2.01 (p = 1.13x10^-8^) after removing "EM35" mouse. The ΔMAP difference between the slopes was 1.53 before (p = 0.00430) and 1.49 (p = 0.00173) after removing “EM35” mouse. The difference in ΔMAPs at GD18 was 13.2 mmHg before (p = 0.00107) and 11.9 mmHg (p = 0.00130) after removal of "EM35" mouse. We obtained similar results when analyzing MAPs (data not shown).

The urinary albumin/creatinine ratio on GD18 was higher in the “EM35” mouse than in other hsFlt-1-e15a-treated mice (**Fig 10B**). Surprisingly, hsFlt-1-e15a expression in the liver was lower in the “EM35” mouse than in other hsFlt-1-e15a-treated mice (**Fig 10C**). Histopathological examination of the liver showed that the “EM35” mouse had multiple cystic biliary hyperplasia and recent and remote thrombotic infarcts in the hepatic parenchyma, suggesting decreased functionality (**Fig 10D–10E**). Aortic ring assays revealed the lowest endothelial outgrowth area in the “EM35” mouse among hsFlt-1-e15a-treated mice (**Fig 10F**). Moreover, mean fetal weights (0.749±0.029g) and placental weights (0.080±0.006g) of the “EM35” mouse were significantly lower than in other hsFlt-1-e15a-treated mice (fetal weight: 1.06±0.023g, p = 2.017x10^-15^, placental weight: 0.111±0.004g, p = 2.05x10^-6^) (**Fig 10G and 10H**). Histopathological examination of the “EM35” mouse placenta showed multiple thrombi in the decidual vessels (**Fig 10I and 10J**).

## Discussion

### Principal findings of this study

The principal findings of this study are as follows: 1) ultrasound predicted pregnancy in 97% of the cases on GD7; 2) ultrasound determined telemetry catheter positions in all cases; 3) the survival rate of the newly implemented, minimally invasive survival cesarean section was 100%; 4) uteri showed complete healing at the incision site on PPD77; 5) hsFlt-1-e15a isoform was mainly expressed in the liver; 6) hsFlt-1-e15a isoform increased mean arterial blood pressure by 12.56 mmHg on GD18 compared to controls (p = 3.9x10^-4^); 7) the mean urine albumin/creatinine ratio on GD18 was five times higher in hsFlt-1-e15a-treated mice than in control mice (p = 0.044); 8) glomerular changes were found in the kidneys of hsFlt-1-e15a-treated mice, including swollen capillary endothelial cells and occlusion of glomerular capillaries; 9) aortic ring assays showed a 46% lesser microvessel outgrowth in hsFlt-1-e15a-treated mice compared to control mice (p = 0.012); 10) there was no difference in placental and fetal weights between the treatment and control groups; and 11) there was one hsFlt-1-e15a-treated mouse that had severe, early-onset preeclampsia-like symptoms associated with fetal growth restriction and multi-organ involvement.

### Pregnancy status was accurately determined with high-resolution ultrasound

Timed-pregnant CD-1 mice arrived on GD5, when only a 75% pregnancy rate was guaranteed by the vendor. Therefore, we used a 55MHz ultrasound probe to determine the pregnancy status of each mouse before the implantation of a telemetry catheter. Initially, ultrasound scans were performed on GD6; however, positive signs of a pregnancy were limited to the detection of gestational sacs ≤2.7mm (**Fig 3A**). Therefore, later we continued the scans on GD7, when an advanced endometrial reaction and embryos were detectable in pregnant uteri (**Fig 3B**). These signs clearly distinguished pregnant uteri from non-pregnant uteri (**Fig 3C**). Among 35 mice scanned on GD7, 32 were correctly diagnosed as pregnant and two as non-pregnant, while one mouse was falsely diagnosed as non-pregnant (**Fig 3D**). Thus, the accuracy of our method was 97% (34/35), showing that pregnancy could be identified with high confidence on GD7. In the case of the mouse with a false negative diagnosis, a very weak endometrial reaction could only be identified without signs of an embryo, which did not match our criteria for the diagnosis of pregnancy. Upon a cesarean delivery, we observed that this mouse had a highly compromised pregnancy and carried only three live fetuses, suggesting that high-frequency ultrasound may be useful for the early detection of pregnancy complications in mice.

### Telemetric blood pressure monitoring provided accurate data acquisition in pregnant mice

Among blood pressure monitoring systems utilized in previous rodent preeclampsia models, the telemetric blood pressure monitoring system offers several advantages over non-invasive systems, including the tail-cuff method [197,199,224]. Telemetric blood pressure monitoring system supports continuous, validated, and reliable data acquisition from unrestricted, conscious mice in their physiological surroundings [225–229]. The telemetric blood pressure monitoring enables the continuous, unstressful detection of circadian blood pressure changes in these very stress-sensitive rodents, and therefore it is the method recommended by the American Heart Association for the continuous blood pressure monitoring in unrestrained animals [230–232]. Here we aimed to implant telemetry catheters at an early stage of gestation so that recovery from surgery would occur before the expected time for blood pressure elevations during the second half of pregnancy. According to our results, the rate of uncomplicated carotid telemetry implantations in pregnant mice [79% (30/38)] were as high as in non-pregnant CD-1 mice in a previous study that compared the complication rates in carotid and abdominal aortic telemetry implantations [233]. The complication rate was higher in the case of the HD-X11 telemetry device [29% (5/17)] than in the case of the TA11PA-C10 transmitter [14% (3/21)]. Among these eight cases with complications, three mice implanted with the TA11PA-C10 devices and one mouse implanted with the HD-X11 device had abnormal body posture and seizures, suggesting ischemic brain damage, while four mice implanted with the HD-X11 devices had transmitter body exteriorization. Because the HD-X11 device is larger in weight and volume [234], the decrease in subcutaneous space around the transmitter and the increase in abdominal circumference during pregnancy could lead to this higher frequency of HD-X11 transmitter exteriorization. These data suggest that the TA11PA-C10 device is more suitable than the HD-X11 device for blood pressure monitoring in pregnant mice.

As a malpositioned telemetry catheter (e.g. a catheter tip remaining in the carotid artery) can lead to thrombotic catheter occlusions and incorrect blood pressure readings [225,233,234], we investigated telemetry catheter positions in all mice. Since the evaluation of catheter positions after euthanization may be biased by accidental dislocation of catheter tips during autopsy, we used high-frequency (55MHz) ultrasound for the accurate, *in vivo* determination of catheter tip positions on GD13 (**Fig 4D**). According to the manufacturer’s recommendation, their positions were treated as optimal when 2mm of the sensing region of the catheter was placed in the aortic arch [234]. These investigations assured us that blood pressure differences between animals did not occur due to malpositioned telemetry catheters.

### A 100% survival rate was obtained with cesarean surgery

Preeclampsia is a syndrome originating from a dysfunctional placenta, since the clinical symptoms usually diminish within a couple of days after the delivery of this organ [11,33–38,42,91,235]. Intriguingly, preeclampsia can also develop postpartum, occurring in ~6% of cases; and thus, the monitoring of the symptoms of preeclampsia is recommended to continue in the early postpartum period [10,17]. Taking into account of these, a major limitation of most mouse preeclampsia models has been that fetuses and placentas were delivered via non-survival surgeries, disabling postpartum monitoring of these animals [197,198,236]. Although some mouse preeclampsia models included postpartum monitoring, in which animals were allowed to deliver naturally [200,237,238], the collection and evaluation of all placentas could have been problematic or impossible in these models, since mice usually deliver during the night and eat their placentas immediately after delivery [239]. Moreover, there is a natural variation in delivery dates of CD-1 pregnant mice according to the information from the vendor, which leads to variation in fetal and placental weights at the time of delivery [240], disabling accurate comparisons of these indices between the study groups.

Because of these facts, we developed a new survival cesarean surgery which enabled follow up on changes in blood pressure and urinary albumin during the postpartum period. In contrast to a previous survival uterine surgery performed in mice [223], we exteriorized only a short segment of one uterine horn at a time, and performed two or three small incisions on each horn (**Fig 5B–5D**). The eight-day survival rate of this surgery was 100% (30/30), and histopathological examinations showed completely healed endometrium, as well as complete healing of the inner layer of the myometrium with only focal disruption of the outer layer at suture sites 77 days after surgery (**Fig 5J–5L**). These results demonstrated that the aseptic, minimally invasive survival cesarean section offers an important advantage for rodent preeclampsia models: namely, the possibility of postpartum blood pressure and urine albumin monitoring after the delivery of fetuses and placentas.

### A full-length, primate-specific sFlt-1-e15a isoform was selected to induce preeclampsia in mice

Fms-like tyrosine kinase-1 (Flt-1) was first characterized in 1990, when Shibuya et al. determined the nucleotide sequence of its encoding cDNA [241–243]. It was revealed that Flt-1 contains seven extracellular Ig-like domains and an intracellular tyrosine kinase domain [241–243] (**Fig 1A**). Later, Flt-1 was shown to bind VEGF and PlGF [242–244] and to be important for embryonic vascular development [243,245]. It was also revealed that the first three extracellular Ig-like domains of Flt-1 are essential for ligand-binding, while the 4–7th extracellular Ig-like domains for receptor dimerization [242,246–248]. In 1993, a soluble isoform of Flt-1 was identified, which is encoded by the first 13 out of 30 exons of *FLT1*, and is generated by skipped splicing of the Flt-1 mRNA and its premature termination due to intron 13 polyadenylation, hence it is denoted as sFlt-1-i13 (**Fig 1B**) [242,243,249,250]. sFlt-1-i13 lacks the tyrosine kinase domain of Flt-1, since it only contains the first six extracellular Ig-like domains, corresponding to 1–657 amino acids in Flt-1, along with a unique 31-amino-acid tail which is encoded by Intron 13 (**Fig 1A**) [242,243,249]. Since this unique tail of sFlt-1-i13 is evolutionarily highly conserved among mammals [242,251], it is thought to have an important biological role [243] (**Fig 1A**). Of importance, sFlt-1-i13 is more abundantly expressed in the placenta than the transmembrane Flt-1 receptor in the second half of the pregnancy in mice [252] and in term gestation in humans [154]. The exact molecular mechanism how the shift from Flt-1 to sFlt-1 production occurs in the placenta is not yet understood [243]. Since sFlt-1-i13 acts as a potent VEGF and PlGF antagonist and a dominant negative inhibitor of angiogenesis [253], it has been suggested to maintain a barrier against extreme VEGF-signaling and vascular hyperpermeability in the placenta [94,243,252].

As an important expansion in the research area, the heterogeneity of human sFlt-1 was described by Thomas et al. in 2007, who discovered a new alternatively spliced sFlt-1 mRNA, which contains the first 14 exon of *FLT1* as well as an alternatively spliced exon (Exon 15a) within an AluSeq retrotransposon, hence it was denoted as sFlt-1-e15a [250,254] (**Fig 1B**). In 2008, Sela et al. published their results on this same sFlt-1 isoform; however, they named it as “sFlt1-14" [94]. Although since then a new terminology was introduced for the sFlt-1 isoforms [153], here we keep with the one described by Thomas et al. reflecting the alternative splicing events during sFlt-1 translation [250,254]. Interestingly, the sFlt-1-e15a mRNA is primate specific, since AluSeq retrotransposons appeared in the primate genome ~40 million years ago [250,254]. The sFlt-1-e15a isoform diverges from Flt-1 after amino acid 706, and contains a unique 28-amino-acid tail (**Fig 1A**) [153]. HsFlt-1-e15a is predominantly expressed in the placenta in humans, and it has a dominant abundance over the hsFlt-1-i13 isoform in the placenta after the first trimester [94,153,154,250,254]. Two additional sFlt-1 isoforms (hsFlt-1-e15b and hsFlt-1-i14) have also recently been described in humans. These are alternatively spliced after exon 14, and contain 13 and 31-amino-acid unique C-termini compared to hsFlt-1-e15a (**Fig 1B**) [153]. These newly described sFlt-1 isoforms have much lower expression in the placenta compared to the two most abundant sFlt-1 isoforms [153,154]. Strikingly, although the transmembrane Flt-1 receptor is the major *FLT1* transcript in most tissues, these four sFlt-1 isoforms account for 95% of all *FLT1* transcripts in the placenta in healthy, term pregnancies [154].

In spite of its important physiological role during pregnancy, sFlt-1-i13 was shown to be overexpressed in the human placenta in preeclampsia [87,255], and it was demonstrated to induce hypertension, proteinuria and glomerular endotheliosis *in vivo* in rats [87]. Since then a plethora of studies have implicated sFlt-1 overexpression in the placenta and in maternal blood in the pathogenesis of preeclampsia [75,76,87,89,90,94,112–116,119,120,122,124,126,129,131,132,135,136,141,144,148]. Later hsFlt-1-e15a expression was found to be up-regulated in the trophoblast by hypoxia [254], and hsFlt-1-e15a isoform to be the most abundant sFlt-1 isoform in the placenta in women with preeclampsia, corresponding to 81.69% of the *FLT1* transcripts [94,154,155]. Based on these facts, since preeclampsia is a primate-specific disease and sFlt-1-e15a is a primate-specific isoform, it was speculated that hsFlt-1-e15a may have yet unidentified biological properties which may be critical in the development of preeclampsia [94,254] Indeed, the unique C-terminus of hsFlt-1-e15a includes a polyserine stretch [94,250], and hsFlt-1-e15a exhibits strong VEGF inhibitor properties [94].

It is important to note that most anti-angiogenic rodent models of preeclampsia utilized adenoviruses overexpressing a truncated sFlt-1 mutant [sFlt-1(1–3)] comprising only 1–329 amino acids from Flt-1 [87,90,196,197,199,201,256–261]. This sFlt-1(1–3) mutant contains the first three Ig-like domains that are involved in VEGF binding, but it neither contains the additional Ig-like domains involved in receptor dimerization nor the unique tail region of sFlt-1-i13 [242]. This artificially truncated sFlt-1(1–3) mutant is naturally not expressed in any species. Therefore, the functional results of these animal studies may not entirely reflect the pathologic conditions in women with preeclampsia. Indeed, a recent mouse model of preeclampsia utilized the full-length, naturally expressed mouse sFlt-1-i13 (msFlt-1-i13) [198], which was less effective in inducing proteinuria than the truncated msFlt-1(1–3) mutant, suggesting that msFlt-1-i13 may have different bioavailability [198]. Another recent study of mice utilized the lentiviral overexpression of human sFlt-1-i13 (hsFlt-1-i13) isoform [200]. This isoform induced late-onset preeclampsia with only a moderate increase in urinary albumin excretion in mice [200].

Based on these previous experiences, we aimed to build a mouse model of preeclampsia in which we can better mimic the full spectrum of the human syndrome. We were interested in testing the *in vivo* pathologic effect of the predominant placental hsFlt-1-e15a transcript in preeclampsia [94,154,155], and whether it can induce the development of preeclampsia in mice.

### Human placental hsFlt-1-e15a expression was most prominent in the liver

Similar to previous studies, our mouse model also utilized adenoviruses overexpressing hsFlt-1-e15a or GFP under the CMV promoter, which was claimed to provide predominant gene expression in the liver [262,263]. In accord with these, we found that hsFlt-1-e15a and GFP mRNA expression was highest in the liver (**Fig 6A and 6B**). Although a viral dose-effect was not seen in GFP expression, a dose-effect was observed in the expression of hsFlt-1-e15a, which had an overall lower expression in all investigated tissues than GFP. These results suggested that the second injection of 1x10^9^ PFU Ad-CMV-hsFlt-1-e15a on GD11 led to a longer and persistent high viral load and hsFlt-1-e15a mRNA expression, and possibly longer persisting biological effects of hsFlt-1-e15a.

Previous studies of preeclampsia utilizing the overexpression of mouse or human sFlt-1-i13 did not aim to detect sFlt-1 protein expression in the placenta [198,200]. Besides the low placental sFlt-1 expression, another explanation may be the lack of suitable antibodies for immunostaining. Indeed, our search failed to find any commercially available antibody which would detect only hsFlt-1-e15a without the cross-reaction with the naturally expressed mouse sFlt-1 variants in the placenta. Because of this limitation, and to confirm the expression of hsFlt-1-e15a and GFP proteins, we infected BeWo human trophoblast-like cells with Ad-CMV-GFP or Ad-CMV-hsFlt-1-e15a, and used a human sFlt-1 specific antibody for Western blots or confocal microscopy for GFP detection. Western blots showed that non-infected BeWo cells expressed low amounts of the 185kDa Flt-1 membrane receptor, and two lower molecular weight (145kDa and 110kDa) sFlt-1 immunoreactive proteins (**S1A Fig**). The size of these protein isoforms corresponded with the 130kDa and 115kDa protein isoforms expressed in HeLa cells by the sFlt1-14 (sFlt-1-e15a) transgene in the study of Sela et al. [94], and the 145kDa and 100kDa sFlt-1 isoforms detected in the plasma of patients with preeclampsia by the study of Rajakumar et al [264], which latter used the same antibody for Western blots as ours [264]. Of interest, Ad-CMV-hsFlt-1-e15a-infected BeWo cells overexpressed both the 145kDa and the 110kDa sFlt-1 variants, which were shown to be glycoproteins of different peptide lengths rather than differentially glycosylated proteins [264]. Indeed, after deglycosylation, the ~145kDa and ~100kDa isoforms became ~80kDa and ~70kDa proteins, respectively [264]. Since the predicted molecular weights of the protein backbones of sFlt-1-i13 and sFlt-1-e15a are 77kDa and 82kDa respectively, it is possible that we and the two other research groups have detected these most abundant isoforms. This can occur if the sFlt-1-i13 isoform can be expressed from the sFlt-1-e15a transgene, which is theoretically possible based on the mode of alternative splicing resulting in this isoform. Confocal microscopy of BeWo cells infected with Ad-CMV-GFP revealed cytoplasmic GFP expression (**S1B Fig**). These results confirmed the functionality of the two adenoviral constructs at the protein level, and pointed out that different human sFlt-1 variants may be secreted into the circulation, as in our mouse model, complicating the blood concentration determinations with conventional techniques.

### Human placental hsFlt-1-e15a increased blood pressure that normalized after delivery

Here we showed that the overexpression of the hsFlt-1-e15a isoform predominantly expressed in the human placenta in preeclampsia can induce blood pressure elevation in mice. Most anti-angiogenic models in rodents mimicked late-onset preeclampsia, with late-gestational blood pressure elevations and mild proteinuria in dams, and without growth restriction of the fetuses [198,260,261]. These models mostly did not include postpartum monitoring, making it impossible to confirm postpartum blood pressure elevation or normalization [87,196,197,199,201,236,261]. Since our model allowed for postpartum monitoring, we monitored blood pressures until PPD7. In the period between GD10 and the cesarean delivery, there was no significant change in blood pressures in the control mice; however, blood pressures significantly increased over time in response to hsFlt-1-e15a treatment, reaching a ΔMAP difference of 13.2 mmHg at parturition (p = 0.00107) (**Fig 7**). After cesarean delivery, ΔMAP decreased in both the control group and in hsFlt-1-e15a-treated mice. However, blood pressures decreased under baseline level in control mice, while these stayed above the baseline in hsFlt-1-e15a-treated mice. These results suggest that the overexpression of hsFlt-1-e15a leads to preeclampsia-like blood pressure elevation in mice, which may stay beyond delivery. The postpartum decrease in blood pressures in hsFlt-1-e15a-treated mice was probably due to the decrease in viral load and hsFlt-1-e15a expression, including the effect of the delivery of the placenta. The delayed normalization of blood pressures may also reflect a conditioning effect of hsFlt-1-e15a on the vasculature.

Previous murine studies employing tail-vein injection of adenoviruses overexpressing sFlt-1 on GD8/GD9 and longitudinal blood pressure monitoring showed peak blood pressure elevations on GD17/GD18, eight to 10 days after adenovirus injection [196–199]. These previous observations were further substantiated by our findings, since mice that received one dose of Ad-CMV-hsFlt-1-e15a on GD8 had their peak MAP on GD18, similar to late-onset preeclampsia in humans. Interestingly, mice that received the second dose of Ad-CMV-hsFlt-1-e15a on GD11 in our study had their peak MAP postpartum (PPD3), similar to late-onset and/or postpartum preeclampsia in humans, suggesting that the extra dose of adenoviruses prolonged—but not heightened—the blood pressure elevation in these animals. This phenomenon suggests that one pathomechanism of postpartum preeclampsia may be that traces of placental tissues producing sFlt-1 and other toxic products may be left in the maternal body.

### Human placental hsFlt-1-e15a impaired endothelial and kidney functions

One of the characteristics of preeclampsia is the generalized dysfunction and damage of the endothelium in the mother, including the morphological and functional changes in glomeruli [8,11,37,77,87,90,106,265,266]. It has been possible to recapitulate these findings in animal models of preeclampsia, especially in those employing the overexpression of anti-angiogenic factors [87,90,198,200], which would lead to a decrease in biological availability of angiogenic factors pivotal to endothelial functions [87,197–200,202,256]. Since aortic ring assays have been previously used to evaluate endothelial functions *in vitro* in some rodent models [220,221,267,268], we established this model to investigate endothelial functions in hsFlt-1-e15a overexpressing mice. We detected a large decrease in endothelial function measured by microvessel outgrowth in hsFlt-1-e15a overexpressing mice compared to controls (46%, p = 0.012) (**Fig 8**). This result suggested that preeclampsia-like symptoms in our mouse model also include endothelial dysfunction, which is a hallmark of preeclampsia in humans [37,77,265].

In most anti-angiogenic models of preeclampsia [87,198,199], glomerular endotheliosis could be detected in the kidneys, consistent with histopathological findings in women with preeclampsia [106,269,270]. In accord, histopathological examinations of the kidneys in this study showed focal glomerular changes in mice overexpressing hsFlt-1-e15a (**Fig 9**). As expected, there were no significant morphological changes in the glomeruli of control mice. We also looked for *in vivo* functional evidence of kidney damage. The determination of the albumin/creatinine ratio for randomly collected human urine specimens has been proposed to replace the inconvenient 24-hour urine collection for the detection and monitoring of microalbuminuria, albuminuria and proteinuria. Although there are limitations of this ratio in clinical use, some animal models of preeclampsia utilized this method recently [90,198,200]. Here we also determined albumin/creatinine ratios in urine samples obtained longitudinally. In accord with the histopathological findings, mean urine albumin/creatinine ratios increased by GD18 and then dropped postpartum in hsFlt-1-e15a overexpressing mice, while these did not change in control mice (**Fig 9**). These results indicated that hsFlt-1-e15a overexpression led to endothelial damage in the kidneys besides endothelial dysfunction in the aorta, suggesting a generalized endothelial dysfunction in the dams.

### Human placental hsFlt-1-e15a induced distinct preeclampsia phenotypes in CD-1 mice

**Fig 11:**
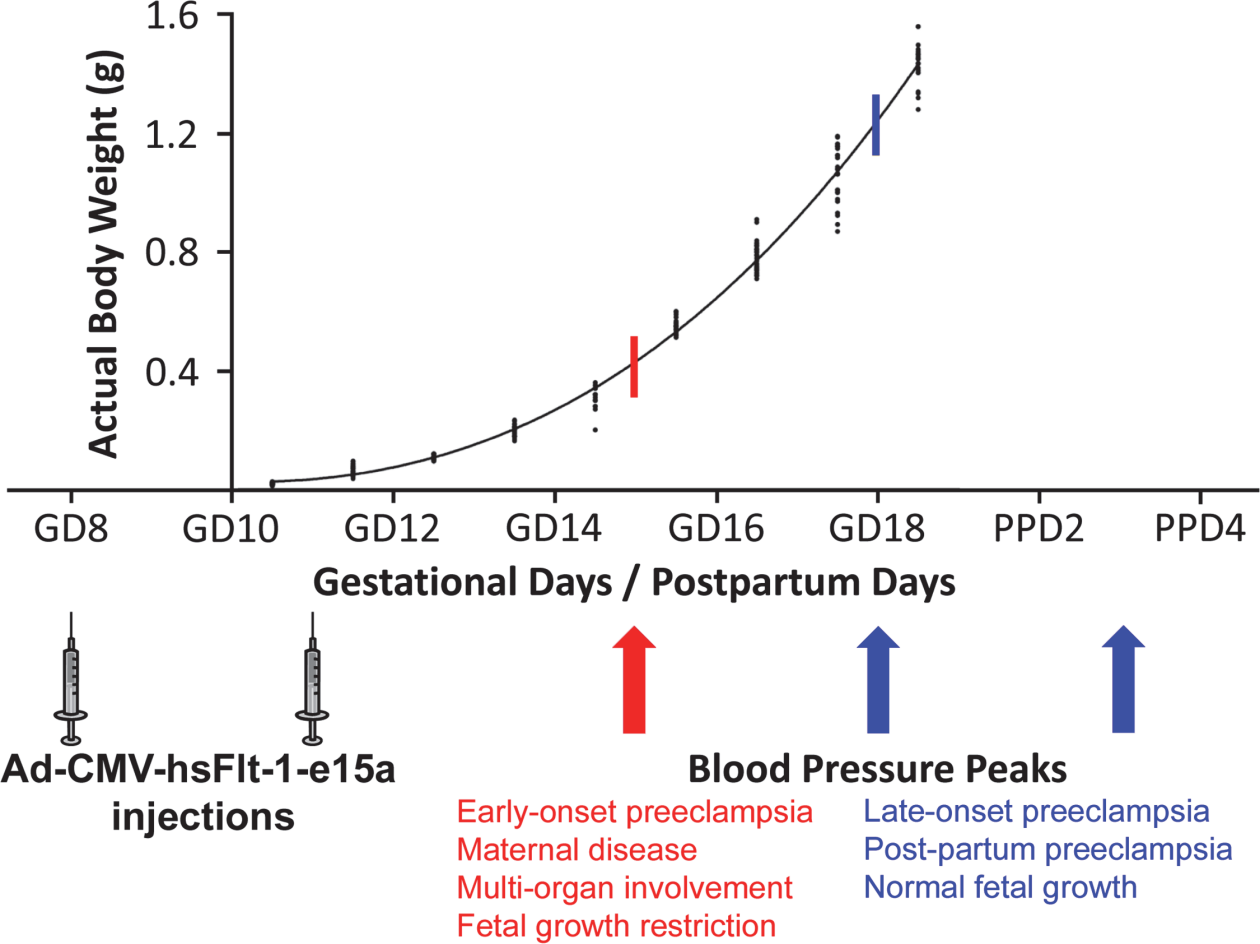
The possible mechanisms of distinct preeclampsia phenotypes induced by hsFlt-1-e15a. Blood pressure peaks (blue arrows) occurred after a ten-day-period following the first and second Ad-CMV-hsFlt-1-e15a injections in all but one mice that developed late-onset or postpartum preeclampsia without growth restriction. In these cases the growth potential of the fetuses was not compromised because they have already reached the top of their growth curve when hsFlt-1-e15a effect reached its plateau (blue stick crossing the curve). In case of “EM35” mouse, blood pressure elevations already peaked at six days after the first Ad-CMV-hsFlt-1-e15a injection (red arrow). In this case the fetal growth potential was severely compromised because the hsFlt-1-e15a effect was the highest when fetuses had reached only about one third or fourth of their growth curve (red stick crossing the curve). Preeclampsia phenotypes are described below the growth curve of CD-1 mouse embryos, which was adapted from a figure in a previous publication of Mu et al. [240]. Permission for reuse of this original figure was obtained from BioMed Central.

Previous anti-angiogenic models of preeclampsia utilizing the overexpression of sFlt-1 in rodents could not recapitulate early-onset preeclampsia associated with fetal growth restriction. We found only a couple of studies where fetal growth restriction was associated with late-onset preeclampsia [90,197,200]. In our study the fetal weights, placental weights, and placental/fetal weight ratios were not affected by hsFlt-1-e15a treatment, and the overexpression of hsFlt-1-e15a in mice mimicked late-onset and postpartum preeclampsia phenotype in humans. The close monitoring of mice during pregnancy and postpartum revealed that blood pressure peaks (GD18 and PPD3) occurred after 10 days following both the first (GD8) and second (GD11) Ad-CMV-hsFlt-1-e15a injections in all mice but one (“EM35”) (**Fig 11**). In these cases, the growth potential of the fetuses was not compromised because they had already reached the top of their growth curve [240] when the hsFlt-1-e15a effect reached its plateau. In the case of the “EM35” mouse, blood pressure elevations peaked at six and seven days after Ad-CMV-hsFlt-1-e15a injections (GD15 and PPD1). In this case, fetal growth restriction was similar to that observed in another mouse model [271]. In the “EM35” mouse, the fetal growth potential was severely compromised because the hsFlt-1-e15a effect was highest when fetuses reached only about one-third or one-fourth of their growth curve. These findings are in good accord with the angiogenic/anti-angiogenic imbalance that develops earlier in pregnancy, and fetal growth restriction occurs more frequently in early-onset preeclampsia than in late-onset preeclampsia [10,75,112,114,116,122,124,131,272].

Our histopathological findings suggested that the “EM35” mouse had cystic biliary hyperplasia, an infrequent but normal background lesion in CD-1 mice according to the information from Charles River Laboratories. As measured by qRT-PCR, this liver disease restricted the expression of hsFlt-1-e15a in the liver, allowing only shorter blood pressure elevations from the 5th to 10th days after Ad-CMV- hsFlt-1-e15a injections. However, this maternal liver disease probably sensitized this animal to any hsFlt-1-e15a effect, and promoted the development of a more severe and fulminant preeclampsia phenotype with heightened and early blood pressure elevation, generalized endothelial dysfunction, and generalized thrombotic disease, which affected the liver and the placenta, and led to consequent fetal growth restriction. We could not investigate maternal coagulation status in this mouse because we did not obtain maternal plasma specimens; however, since CD-1 is an outbred mouse strain with individual animals having a different genetic background [273–275], we hypothesize that the “EM35” mouse might have had a thrombotic disorder. This finding is consistent with the observation that inherited and acquired thrombophilias predispose for the development of severe preeclampsia and early-onset preeclampsia in humans [276–279], and also that women developing early-onset preeclampsia may have a pre-existing metabolic disease [22,280]. Of importance, among previous mouse models of preeclampsia, those which involved mice that had a maternal knock-out phenotype [e.g. eNOS(-/-), IL10(-/-), C1q(-/-)] were able to generate severe preeclampsia phenotype associated with intrauterine growth restriction [85,202,238,281]. Based on these human and experimental animal data it seems that the phenotype of preeclampsia is fundamentally influenced by both fetal (placental factors released to the maternal circulation) and maternal (genetic and environmentally induced diseases) factors, and the parallel existence of certain maternal diseases to preeclampsia can promote the development of an early-onset preeclampsia phenotype.

## Conclusions

A mouse model of late-onset preeclampsia was first developed with the overexpression of hsFlt-1-e15a, verifying the *in vivo* pathologic effects of this primate-specific, predominant placental sFlt-1-e15a isoform. Interestingly, hsFlt-1-e15a induced early-onset preeclampsia-like symptoms associated with IUGR in a mouse with a liver disease. Our findings support that hsFlt-1-e15a is central to the terminal pathway of preeclampsia, and it can induce the full spectrum of symptoms in this severe obstetrical syndrome.

### Dedication

This manuscript is dedicated to the memory of the late Mary King, whose professionalism and wonderful personality will be forever remembered by her colleagues.

## Supporting Information

S1 Fig Profiling of hsFlt-1-e15a and GFP expression. (A) Western blot shows the expression of the 185kDa transmembrane Flt-1 receptor as well as 145kDa and 110kDa sFlt-1 variants in human BeWo trophoblast-like cells not infected with adenovirus (Ctr), infected with Ad-CMV-GFP (GFP), or infected with Ad-CMV-hsFlt-1-e15a. Ad-CMV-hsFlt-1-e15a enhances the overexpression of 145kDa and 110kDa sFlt-1 variants in BeWo cells. MW markers are depicted in the left; Flt-1 and sFlt-1 variants are depicted on the right. (B) A confocal microscopic image shows cytoplasmic GFP expression in BeWo cells infected with Ad-CMV-GFP. (C) Control BeWo cells not infected with adenovirus. (B-C) 2000x magnifications.
(TIF)

S2 Fig Blood pressure monitoring of day and night cycles. X-axes show gestational days (GDs) and postpartum days (PPDs). Mean arterial pressure changes (ΔMAP) are depicted on the Y-axes. Red and blue dots and standard error bars represent day and night ΔMAP values for given time-points, respectively. Red and blue lines depict the ΔMAP patterns during day and night cycles, respectively. ΔMAP was 2.67 mmHg higher during the night cycles than the day cycles (p = 2.5x10^-3^) before cesarean delivery, and it was 4.37 mmHg higher during the night cycles than the day cycles (p = 2.7x10^-6^) after cesarean delivery.
(TIF)

S3 Fig Blood pressure monitoring and mean arterial pressures. X-axes show gestational days (GDs) and postpartum days (PPDs). Mean arterial pressures (MAPs) are depicted on the Y-axes. Prior to parturition, there was no change in the blood pressures over time in control mice (MAP slope = 0.426 mmHg/day; p = 0.174) (A,C). However, in response to hsFlt-1-e15a treatment, blood pressures increased over time (MAP slope = 2.02 mmHg/day; p = 1.13x10^-10^). The MAP slope in the hsFlt-1-e15a-treated groups was higher compared to the controls (1.59 mmHg/day; p = 2.6x10^-4^) (B,D). The estimated effect of hsFlt-1-e15a treatment on GD18 was 6.90 mmHg (p = 0.0339) in MAP.
(TIF)

S4 Fig Placental weights, fetal weights and placental/fetal weight ratios. (A) Placental weights [mean±2 standard error (SE)] were not different between the groups (GFP 1x: 0.109±0.009g; hsFlt-1-e15a 1x: 0.117±0.015; GFP 2x: 0.098±0.014g; hsFlt-1-e15a 2x: 0.102±0.010g). (B) Fetal weights (mean ±2SE) were not different between the groups (GFP 1x: 1.03±0.097g; hsFlt-1-e15a 1x: 1.08±0.059g; GFP 2x: 1.07±0.064g; hsFlt-1-e15a 2x: 0.102±0.010g). (C) Placental/fetal weight ratios (mean±2SE) were not different between the groups (GFP 1x: 0.107±0.014g; hsFlt-1-e15a 1x: 0.109±0.017g; GFP 2x: 0.092±0.009g; hsFlt-1-e15a 2x: 0.102±0.008g).
(TIF)
