# Supplementary figures and images for: Full-Length Human Placental sFlt-1-e15a Isoform Induces Distinct Maternal Phenotypes of Preeclampsia in Mice
Source: PLoS One. 2015 Apr 10;10(4):e0119547. doi: 10.1371/journal.pone.0119547 (PMC4393117; doi:10.1371/journal.pone.0119547)

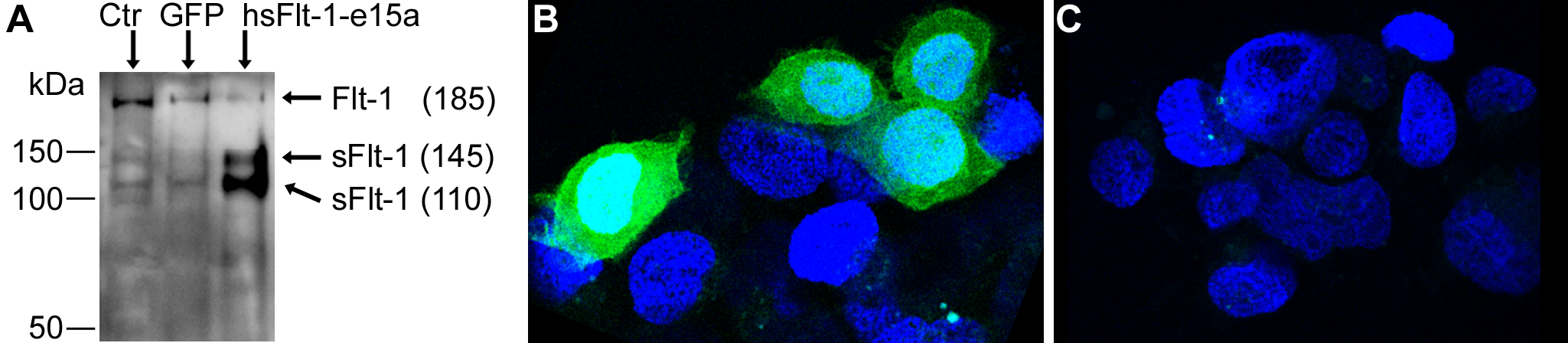

Supplement: S1 Fig — (A) Western blot shows the expression of the 185kDa transmembrane Flt-1 receptor as well as 145kDa and 110kDa sFlt-1 variants in human BeWo trophoblast-like cells not infected with adenovirus (Ctr), infected with Ad-CMV-GFP (GFP), or infected with Ad-CMV-hsFlt-1-e15a. Ad-CMV-hsFlt-1-e15a enhances the overexpression of 145kDa and 110kDa sFlt-1 variants in BeWo cells. MW markers are depicted in the left; Flt-1 and sFlt-1 variants are depicted on the right. (B) A confocal microscopic image shows cytoplasmic GFP expression in BeWo cells infected with Ad-CMV-GFP. (C) Control BeWo cells not infected with adenovirus. (B-C) 2000x magnifications. (TIF) [file pone.0119547.s001.tif]

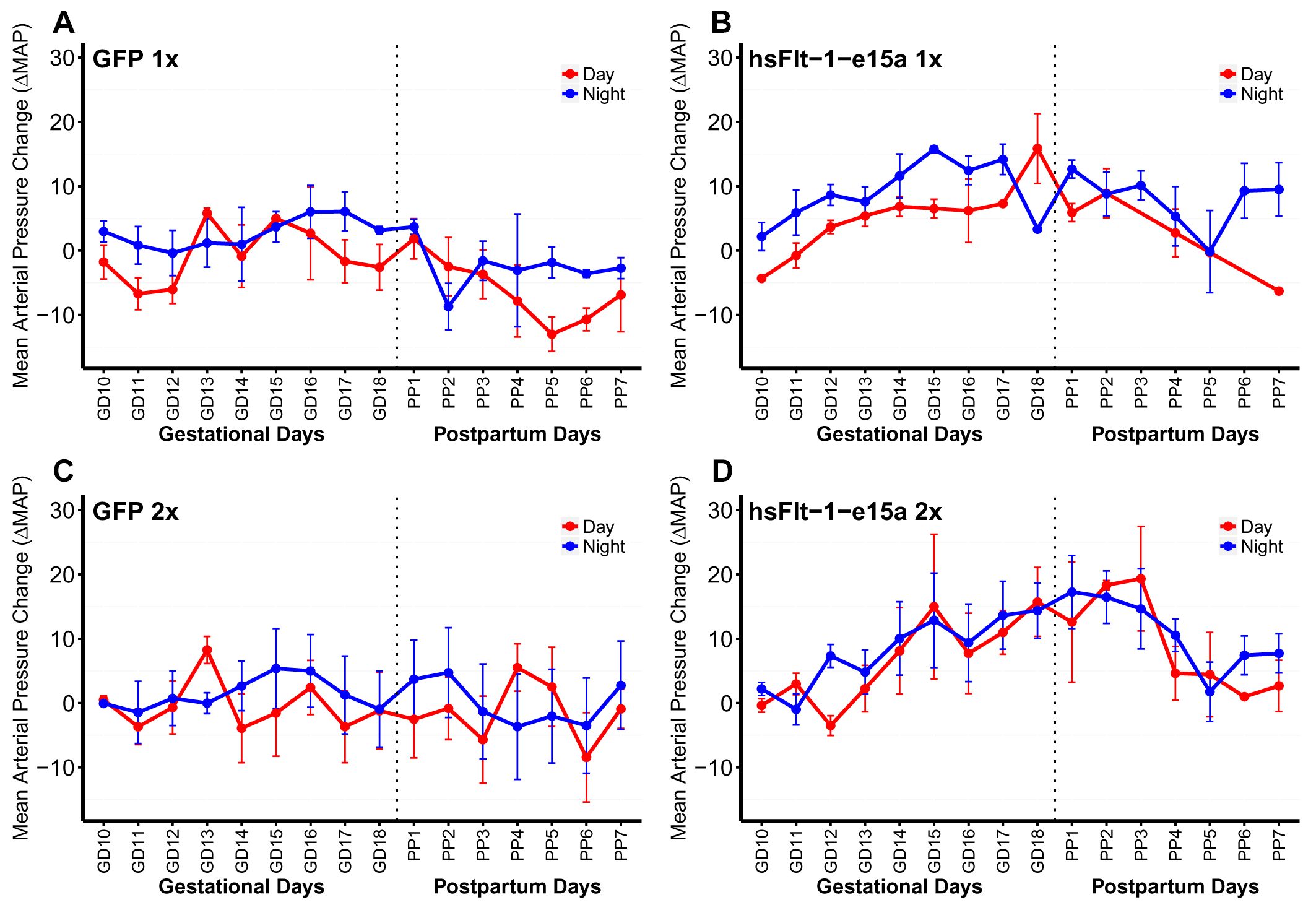

Supplement: S2 Fig — X-axes show gestational days (GDs) and postpartum days (PPDs). Mean arterial pressure changes (ΔMAP) are depicted on the Y-axes. Red and blue dots and standard error bars represent day and night ΔMAP values for given time-points, respectively. Red and blue lines depict the ΔMAP patterns during day and night cycles, respectively. ΔMAP was 2.67 mmHg higher during the night cycles than the day cycles (p = 2.5x10^-3^) before cesarean delivery, and it was 4.37 mmHg higher during the night cycles than the day cycles (p = 2.7x10^-6^) after cesarean delivery. (TIF) [file pone.0119547.s002.tif]

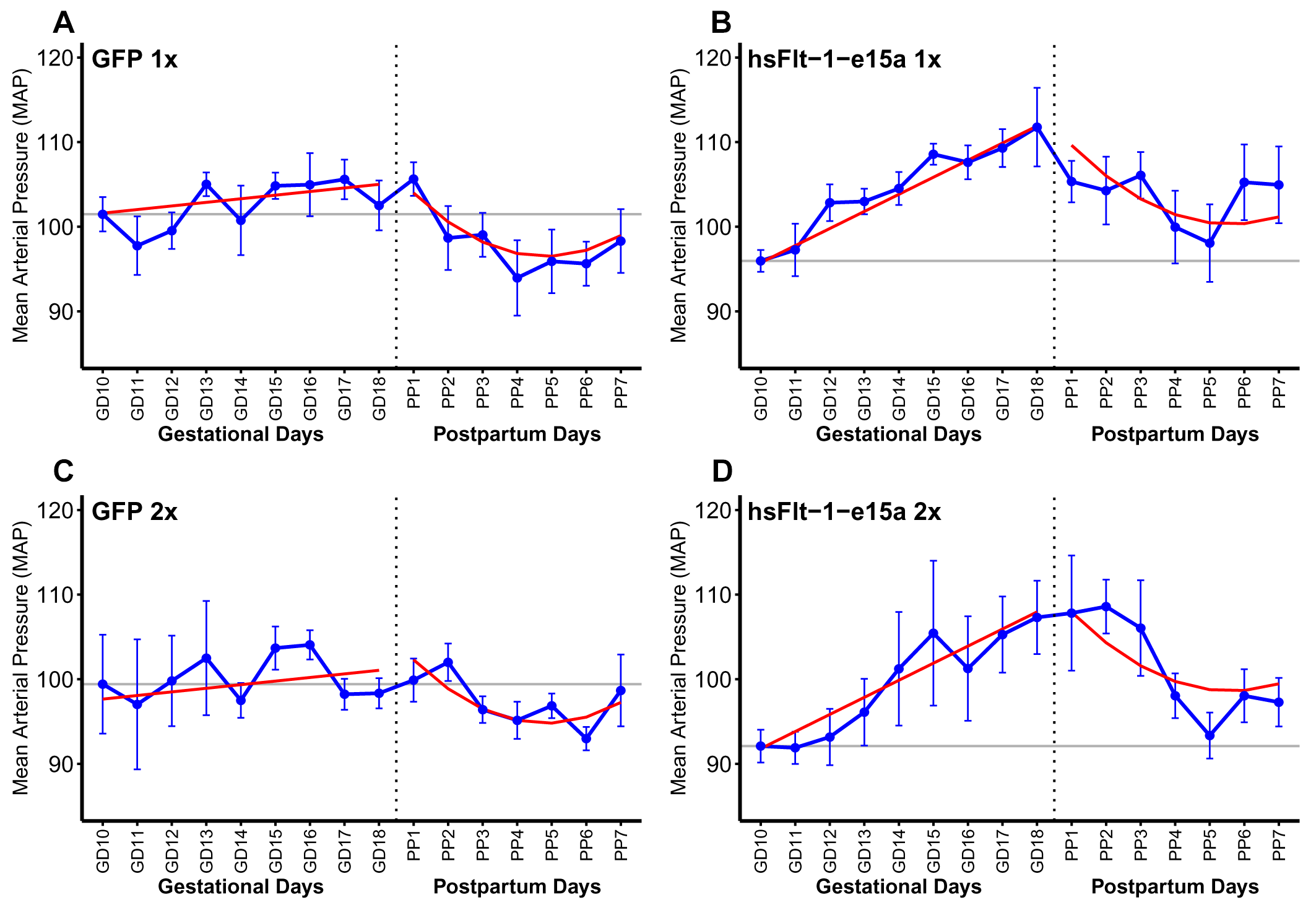

Supplement: S3 Fig — X-axes show gestational days (GDs) and postpartum days (PPDs). Mean arterial pressures (MAPs) are depicted on the Y-axes. Prior to parturition, there was no change in the blood pressures over time in control mice (MAP slope = 0.426 mmHg/day; p = 0.174) (A,C). However, in response to hsFlt-1-e15a treatment, blood pressures increased over time (MAP slope = 2.02 mmHg/day; p = 1.13x10^-10^). The MAP slope in the hsFlt-1-e15a-treated groups was higher compared to the controls (1.59 mmHg/day; p = 2.6x10^-4^) (B,D). The estimated effect of hsFlt-1-e15a treatment on GD18 was 6.90 mmHg (p = 0.0339) in MAP. (TIF) [file pone.0119547.s003.tif]

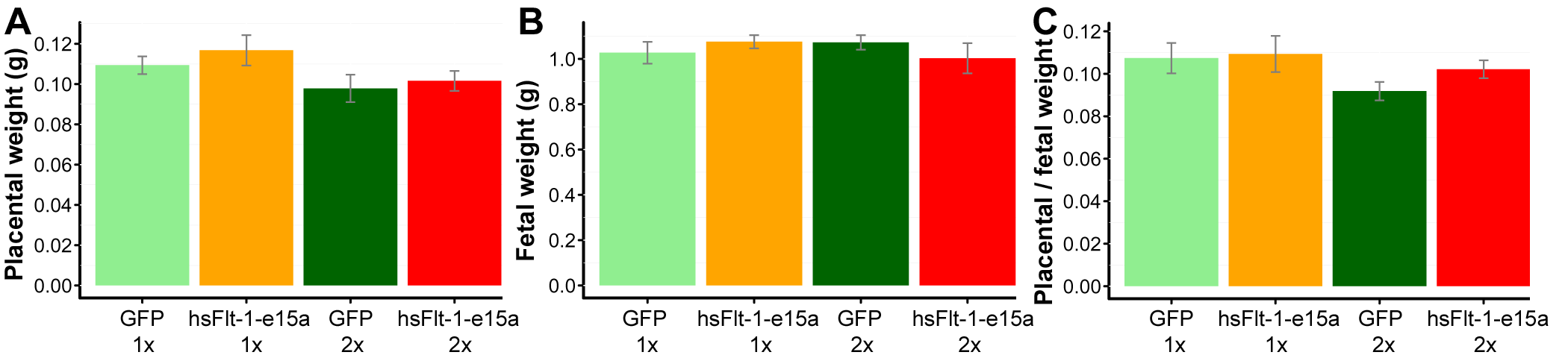

Supplement: S4 Fig — (A) Placental weights [mean±2 standard error (SE)] were not different between the groups (GFP 1x: 0.109±0.009g; hsFlt-1-e15a 1x: 0.117±0.015; GFP 2x: 0.098±0.014g; hsFlt-1-e15a 2x: 0.102±0.010g). (B) Fetal weights (mean ±2SE) were not different between the groups (GFP 1x: 1.03±0.097g; hsFlt-1-e15a 1x: 1.08±0.059g; GFP 2x: 1.07±0.064g; hsFlt-1-e15a 2x: 0.102±0.010g). (C) Placental/fetal weight ratios (mean±2SE) were not different between the groups (GFP 1x: 0.107±0.014g; hsFlt-1-e15a 1x: 0.109±0.017g; GFP 2x: 0.092±0.009g; hsFlt-1-e15a 2x: 0.102±0.008g). (TIF) [file pone.0119547.s004.tif]
